# A widely-applicable high-throughput cellular thermal shift assay (CETSA) using split Nano Luciferase

**DOI:** 10.1038/s41598-018-27834-y

**Published:** 2018-06-21

**Authors:** Natalia J. Martinez, Rosita R. Asawa, Matthew G. Cyr, Alexey Zakharov, Daniel J. Urban, Jacob S. Roth, Eric Wallgren, Carleen Klumpp-Thomas, Nathan P. Coussens, Ganesha Rai, Shyh-Ming Yang, Matthew D. Hall, Juan J. Marugan, Anton Simeonov, Mark J. Henderson

**Affiliations:** National Center for Advancing Translational Sciences, National Institutes of Health, Rockville, Maryland 20850 USA

## Abstract

Assessment of the interactions between a drug and its protein target in a physiologically relevant cellular environment constitutes a major challenge in the pre-clinical drug discovery space. The Cellular Thermal Shift Assay (CETSA) enables such an assessment by quantifying the changes in the thermal stability of proteins upon ligand binding in intact cells. Here, we present the development and validation of a homogeneous, standardized, target-independent, and high-throughput (384- and 1536-well formats) CETSA platform that uses a split Nano Luciferase approach (SplitLuc CETSA). The broad applicability of the assay was demonstrated for diverse targets, and its performance was compared with independent biochemical and cell-based readouts using a set of well-characterized inhibitors. Moreover, we investigated the utility of the platform as a primary assay for high-throughput screening. The SplitLuc CETSA presented here enables target engagement studies for medium and high-throughput applications. Additionally, it provides a rapid assay development and screening platform for targets where phenotypic or other cell-based assays are not readily available.

## Introduction

Small molecules that modulate protein function are useful as tools for interrogating biological processes and as therapeutic agents. Compounds with high affinity and specificity for a target are often discovered using *in vitro* assays incorporating purified proteins. However, maintaining desirable on-target activity of a small molecule when transitioning from a biochemical to a cellular environment can be a challenge. Loss of activity can be attributed to many factors including low cell permeability, off-target protein binding, compound efflux, or a protein’s structure/complexation/accessibility in the native environment^1–3^. Experimental approaches that account for variables found in complex cellular environments are argued to improve confidence in mechanism of action. In some cases, phenotypic assays (*e.g*. cell death, calcium flux, reporter genes) are used to assess small molecule activity in cells; however, these are prone to artifacts driven by off-target or “indirect” effects^4^. Alternatively, direct evidence of small molecule binding in living systems can be pursued with target engagement studies, for example using radioligand competition assays^5^. More recently, fluorescent probes have been utilized, which are detected when in close proximity to bioluminescent (BRET) or fluorescently labeled (FRET-FLIM) targets^6,7^. Alternatively, modification of small molecules with reactive groups (*e.g*. electrophilic trap or photocrosslinking group) can reveal target engagement when coupled with covalent entrapment, affinity purification and mass spectrometry^8,9^. While extremely valuable for confirming target engagement in living systems, these experiments require context-specific modification of compounds and/or proteins, which ultimately limits universal utilization and throughput.

The Cellular Thermal Shift Assay (CETSA) has revolutionized cell-based target engagement studies as it allows binding to be examined in cells using unmodified compounds^10^. The assay is based on the premise that upon heating, a protein will unfold and aggregate at a given temperature, described as the T_agg_ (or T_m_)^11^. Heat-induced aggregation can be altered by a small molecule binding to the protein, causing a shift in T_agg_/T_m_ (also referred to as thermal shift)^12^. Ligand-induced thermal shifts have been studied extensively using purified proteins, where changes in T_m_ are most commonly detected by differential scanning fluorimetry using hydrophobic dyes^13^ or intrinsic fluorescence of tryptophan residues^14^. The biophysical principles of thermal unfolding are retained for CETSA, albeit in the complex environment of intact cells. After heating cells in the presence of a small molecule, the unfolded and aggregated proteins are removed by centrifugation, and thermal stability of individual proteins that remain in solution can be monitored by a protein detection method such as western blot. More recently, mass spectrometry has been utilized for detection of a substantial portion of the entire melting proteome^15^.

To support drug screening efforts, alternative detection modalities have been implemented to increase the throughput of CETSA. Almqvist *et al*. reported the coupling of CETSA and AlphaLISA to screen for molecules that bind thymidylate synthase^16^, and a similar strategy was recently applied to screen for androgen receptor binders^17^. However, the AlphaLISA methodology requires the development and optimization of reagents (*e.g.* target-specific antibody pairs) and methods unique for each target. Towards a more universal detection approach, several groups have described the use of an enzyme fragment complementation (EFC) system where a small fragment (42 amino acid) of β-galactosidase is tagged to the target of interest, and compound-mediated target stabilization is subsequently detected by addition of the enzyme acceptor (EA) fragment. In the initial iterations of this system, compound binding was detected as a protein stabilization event under physiologic conditions, as demonstrated for MEK1^18^, PRMT3^19^, and BRD4^20^. More recently, the approach was adapted to a CETSA format to profile 123 SMYD3 inhibitors for thermal stabilization^21^. To date, a 1,536-well compatible CETSA assay able to support ultra-high-throughput drug screening campaigns has not been reported.

We have successfully developed a target-independent CETSA platform that is compatible with both 384- and 1,536-well formats using a split NanoLuciferase (SplitLuc) reporter approach. A 15-amino acid tag from NanoLuc was appended to a set of proteins that span multiple functions, sizes, structures, and subcellular localizations. The small tag was selected to minimize the potential impact on protein function, and complementation with the large fragment of NanoLuc provides a luminescence reporter that compares favorably to other complementation strategies. Performance of the SplitLuc CETSA assay was assessed relative to independent biochemical and cell-based assays using a set of well-characterized inhibitors. Compatibility with high-throughput screening was highlighted by profiling a cancer-focused library and kinase inhibitor library for binders of LDHA and CDK9, respectively. Our results indicate that the SplitLuc CETSA approach facilitates target engagement studies for medium to ultra-high-throughput applications. Additionally, it provides a rapid assay development and screening platform for targets where phenotypic or other cell-based assays are not readily available.

## Results

**Figure 1:**
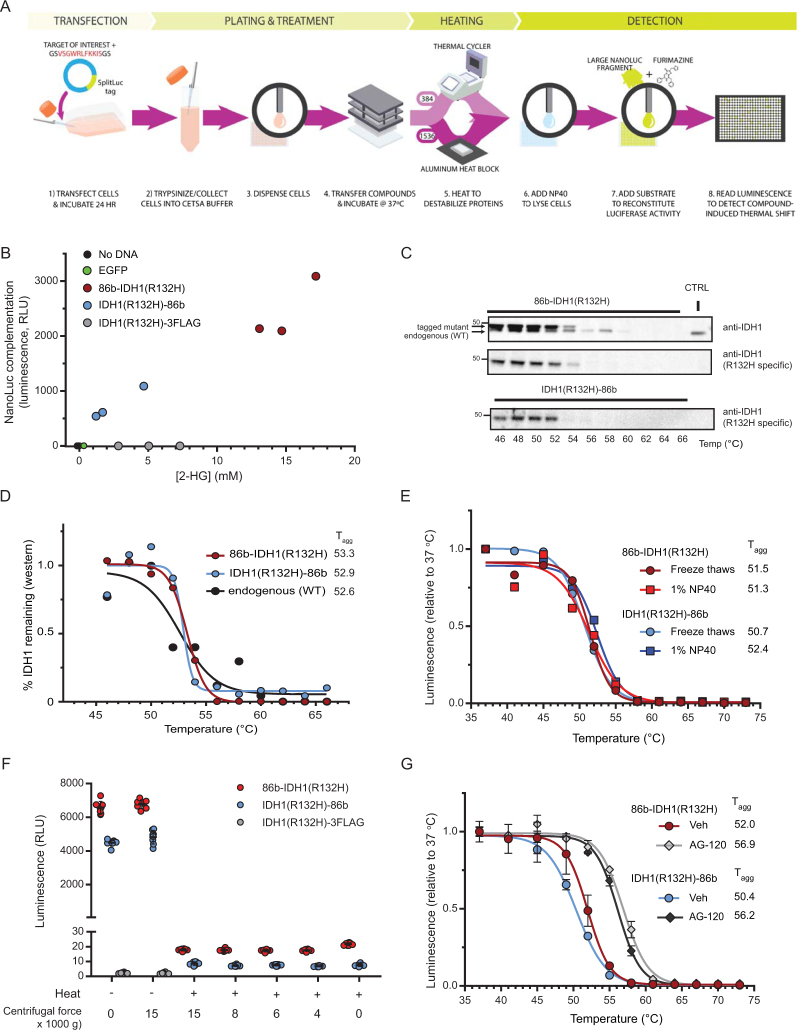
SplitLuc CETSA assay development. (**A**) Schematic overview of the ultra-high-throughput cellular thermal shift assay using split NanoLuc. A peptide (86, red text) previously reported as having strong affinity for a large fragment of NanoLuc (11S) was modified to have Gly-Ser linkers on each side (86b-tag). (**B**) 2-HG production in cells expressing tagged IDH1(R132H) or controls (EGFP or no DNA). 86b was fused to either the N- or C-terminus of IDH1(R132H). As a control for the 86b tag a FLAG tag was fused to the C-terminus of IDH1(R132H). Enzymatic activity of the tagged transgene was assessed by measuring 2-HG in the medium 72 h post-transfection (n = 3 per variant, x-axis). Transgene expression levels were measured by luminescence resulting from NanoLuc complementation (y-axis). (**C**) Thermal stability of endogenous (wild-type) and transfected (R132H mutant) IDH1 by immunoblot using pan (top) or R132H specific (middle, N-tag; lower, C-tag) antibodies. Untransfected/unheated HEK293T cells (endogenous WT IDH1) serve as the control (CTRL) sample. (**D**) Densitometric analysis of immunoblot in (**C**) enables quantification of T_agg_ for endogenous and 86b-tagged IDH1(R132H). (**E**) Thermal profiles of 86b-IDH1(R132H) and IDH1(R132H)-86b extracted via freeze-thaw or NP40-mediated lysis. (**F**) Aggregated IDH1(R132H) is complementation incompetent and does not require removal by centrifugation. After heating to 65 °C for 3.5 min, samples were centrifuged at various speeds for 20 min and complementation was assessed. (**G**) Treatment with 1 µM AG-120, an inhibitor of mutant IDH1, stabilizes N- and C-tagged IDH1(R132H). Cells were treated for 1 h and heated for 3.5 min (mean ± SD, n = 3).

In order to provide target engagement data to support decision-making for several of our small molecule screening programs, we set out to develop a CETSA approach that would be compatible with a high-throughput 1,536-well format. Additionally, our goal was to implement a modular strategy, in which the assay format and procedures would be easily adaptable to diverse targets. Towards these goals, we developed an enzyme complementation assay by appending a small split-NanoLuc peptide to protein targets (Fig. 1A). Previous work identified an 11-amino acid peptide referred to as “#86” (also known as HiBiT^22^), as part of a split-NanoLuc pair, with a sub-nanomolar dissociation constant for a large fragment of the enzyme (11S)^23^. Gly-Ser linkers were added to each end of the #86 peptide, to create a 15-amino acid tag, hereafter referred to as 86b. With purified components, complementation-dependent luminescence was linear for 86b ranging from 10 pM to 60 nM upon addition of 100 nM 11S (Supplementary Fig. S1A). Next we measured reconstituted NanoLuc activity in cells by transiently transfecting constructs expressing 86b-tagged target into the universally-used human embryonic kidney cell line HEK293T. This methodology bypasses the need for generating stable cell lines or specialized target-specific transduction regents (such as BacMam), thus significantly streamlining the assay. We initially focused on mutant IDH1(R132H) as a proof-of-concept target, for which we have recently developed a pre-clinical drug development assay suite, including a CETSA with western blot detection^24^. Reconstituted luciferase activity was detected in lysates from cells transfected with 86b-tagged IDH1(R132H), but not a FLAG-tagged control (Supplementary Fig. S1B). The production of 2-hydroxyglutarate (2-HG) was elevated by IDH1(R132H) overexpression, indicating the peptide tag did not abrogate the neomorphic enzymatic activity of IDH1(R132H) (Fig. 1B). The thermal melt profiles for both N- and C-tagged IDH1(R132H) were similar to that of endogenous wild-type (WT) IDH1 when assessed by Western blot analysis (Fig. 1C,D; Supplementary Fig. S1C) and NanoLuc complementation (Supplementary Fig. S1D), in accordance with previous findings using circular dichroism^25^. The luminescence signal produced as a result of NanoLuc complementation increased with cell density and an optimal signal-to-background ratio was observed for samples containing 1 × 10^6^ cells/mL (Supplementary Fig. S1E). Traditional CETSA methodology includes freeze-thaw and centrifugation steps to lyse cells and allow removal of insoluble proteins, respectively. Such manipulations present challenges for a high-throughput workflow, so alternative methods were developed. Cell lysis was achieved by addition of 1% Nonidet P-40 (NP40), which was an acceptable substitution for freeze-thaw cycles (Fig. 1E). Importantly, NP40 did not disrupt 86b/11S complementation at concentrations below 2% (Supplementary Fig. S1F). The emission of luminescence peaked at about 10 minutes after substrate addition and decayed with a half-life of 29 minutes (Supplementary Fig. S1G). The path to developing a high-throughput amenable assay was further simplified after demonstrating that centrifugation was not required to remove aggregated IDH1(R132H)-86b after heating, allowing the assay to be performed without a separation step (Fig. 1F). We next examined compound-mediated stabilization of tagged IDH1(R132H), and observed a T_agg_ shift of ~5 °C after treatment with the inhibitor AG-120 (Fig. 1G). Stabilization was observed in each of four human cell lines examined (Supplementary Fig. S1H) and also when the compound was added directly to cellular lysate (Supplementary Fig. S1I).

**Figure 2:**
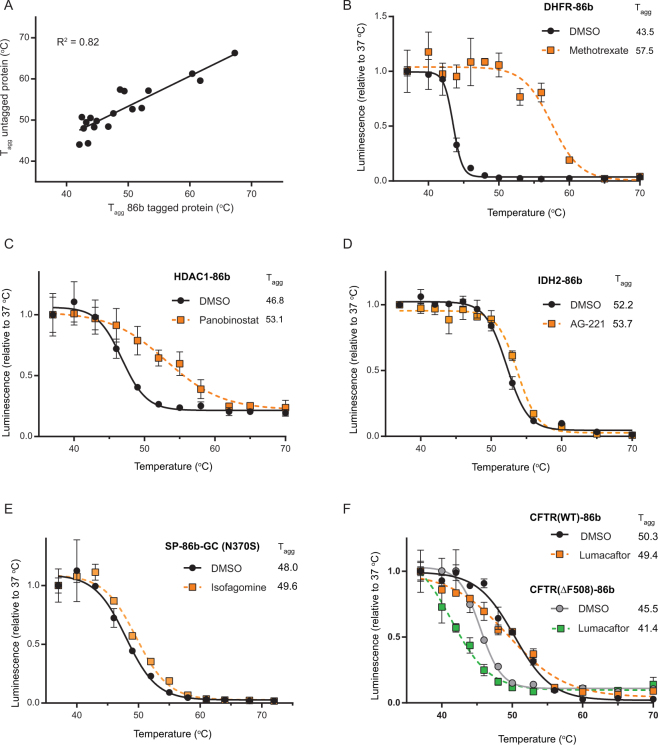
CETSA approach for targets of diverse function and subcellular localizations. (**A**) T_agg_ for a set of eighteen 86b-tagged constructs (all C-terminal placement) was compared to endogenous protein T_agg_, as reported in a thermal proteome profiling dataset. (**B**–**F**) Thermal melt profiles for (**B**) DHFR, (**C**) HDAC1, (**D**) IDH2 [95% CI T_agg_: Veh (51.9, 52.4); AG-221 (53.3, 54.1)], (**E**) glucocerebrosidase [95% CI T_agg_: Veh (46.9, 49); isofagomine (49, 50.4)], and (**F**) cystic fibrosis transmembrane conductance regulator. For each transgene, thermal stabilization was tested after 1 h treatment with 20 µM indicated compound (mean+/−SD, n = 3).

The broader applicability of the SplitLuc CETSA was demonstrated by examining additional targets diverse in structure, function, and subcellular localization (Supplementary Table S1). First, we compared the melting temperatures of eighteen 86b-tagged constructs to those of their endogenous counterparts, which were reported in a thermal proteome profiling dataset^15^. Overall, the T_agg_ of endogenous and 86b-tagged targets showed good agreement (Fig. 2A). Next, compound-induced thermal stabilization was examined for a subset of targets using known small molecule modulators of target activity. The cytosolic enzyme dihydrofolate reductase (DHFR-86b) was robustly stabilized by Methotrexate, in agreement with previous reports^10^ (Fig. 2B). Nuclear histone deacetylase 1 (HDAC1-86b) was stabilized by the HDAC inhibitor Panobinostat (Fig. 2C). For some nuclear targets, recovery was enhanced by incorporating a high-salt extraction step (Supplementary Fig. S2B,C). AG-221, an allosteric modulator of the mitochondrial enzyme IDH2, induced a small, but significant, T_agg_ shift in IDH2-86b (Fig. 2D) and similarly stabilized both WT and R172K enzymes (Supplementary Fig. S2A). For the mutant lysosomal protein glucocerbrosidase (GBA) N370S, the iminosugar chaperone isofagomine induced a T_agg_ shift of 1.6 °C (Fig. 2E). Lumacaftor, a recently FDA-approved chemical chaperone indicated for cystic fibrosis, was tested against the cystic fibrosis transmembrane conductance regulator CFTR, which is localized to the endoplasmic reticulum and plasma membrane. Both the WT-CFTR-86b and pathogenic ΔF508-CFTR-86b proteins were assessed (Fig. 2F). As expected, the thermal stability of ΔF508 CFTR was lower than that of the WT protein (T_agg_ shift of −4.8 °C). Although Lumacaftor destabilized both proteins, it had a more pronounced destabilization effect on the pathogenic mutant, with a T_agg_ shift of −4.1 °C. While destabilization is perhaps unexpected for a chemical chaperone, our observations are in agreement with a recent report that used differential scanning calorimetry to show that direct binding of Lumacaftor to CFTR induces thermal destabilization, indicative of compound binding to a non-native state^26^. Altogether, our initial results indicated that the SplitLuc approach constitutes a homogenous reporter-based assay that could be adopted for a wide-range of targets and streamlined for high-throughput quantification of ligand-mediated protein stabilization (or destabilization).

**Figure 3:**
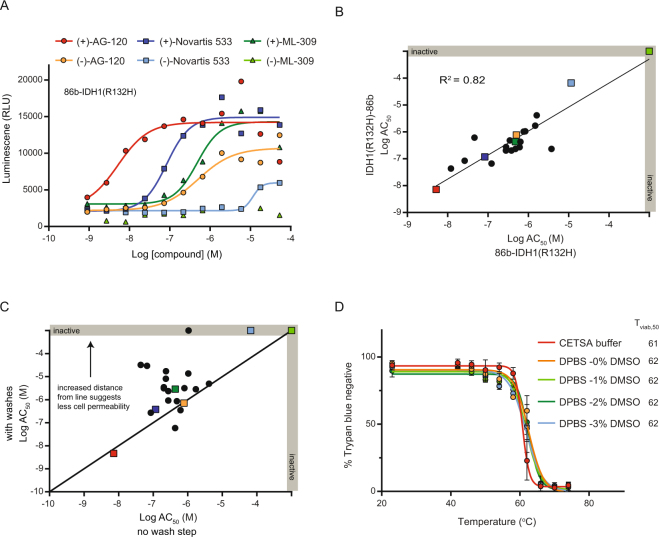
Assay miniaturization to a 384-well format. (**A**) Cells expressing N-tagged IDH1(R132H) were treated with three pairs of IDH1 inhibitors, previously characterized as the more active eutomer (+) or less active distomer (−), and heated at 56  °C for 3.5 min. (**B**) Correlation plot for the N- and C-tagged variants comparing AC_50_ for thermal stabilization. Compounds presented in panel A are indicated by colored squares. (**C**) The potencies of many compounds were reduced with a wash step introduced before heating, as indicated by upward shift from black line (x = y). Some compounds, *e.g*. AG-120 (red square), did not show a shift in AC_50_. (**D**) HEK293T cell permeability during the heating step was examined using a Trypan Blue exclusion assay in CETSA buffer containing 0 to 3% DMSO (mean+/−SD, n = 2 counts per group; avg. 183 cells per count).

Miniaturization of the SplitLuc assay to a 384-well format was examined with the mutant IDH1(R132H) construct and a set of well-characterized inhibitors. Three mutant IDH1 inhibitors stabilized proteins tagged at both the N- and C-terminus, with the eutomers showing more potent effects than the corresponding distomers (Fig. 3A and Supplementary Fig. S3A). For nine compounds that were tested in both the SplitLuc and traditional CETSA using immunoblot, we observed a good correlation in thermal stabilization potency (Supplementary Fig. S3B). For a larger set of 23 inhibitors, a strong correlation for both potency (Fig. 3B; AC_50_, the concentration at which half-maximal activity was observed^27^) and overall stabilization profile (Supplementary Fig. S3C–E; potency and max response captured using area under the curve^28^) was observed for the N- and C-tagged proteins. Similar results were observed for the 23 inhibitors when the assay was performed by two independent users (Supplementary Fig. S3F).

Washing cells prior to heating decreased the apparent potency for some compounds (Fig. 3C), suggesting membrane integrity may be disrupted during the heating phase, thus allowing compound to engage targets prior to aggregation. A Trypan Blue exclusion assay demonstrated that HEK293T membrane integrity was unaffected up to ~55 °C with no effect by the vehicle DMSO at concentrations up to as 3% (Fig. 3D). The heating temperature of 56 °C used for IDH1(R132H) was in a range where membrane permeability could reasonably account for the observed wash-induced shift; however, an independent measurement of compound permeability (PAMPA) showed no correlation with changes in potency, suggesting additional contributing factors (Supplementary Fig. S3G). A similar profile for temperature versus Trypan Blue exclusion was observed for six additional cell lines, with permeabilization induced between 57 and 63 °C (Supplementary Fig. S3H).

**Figure 4:**
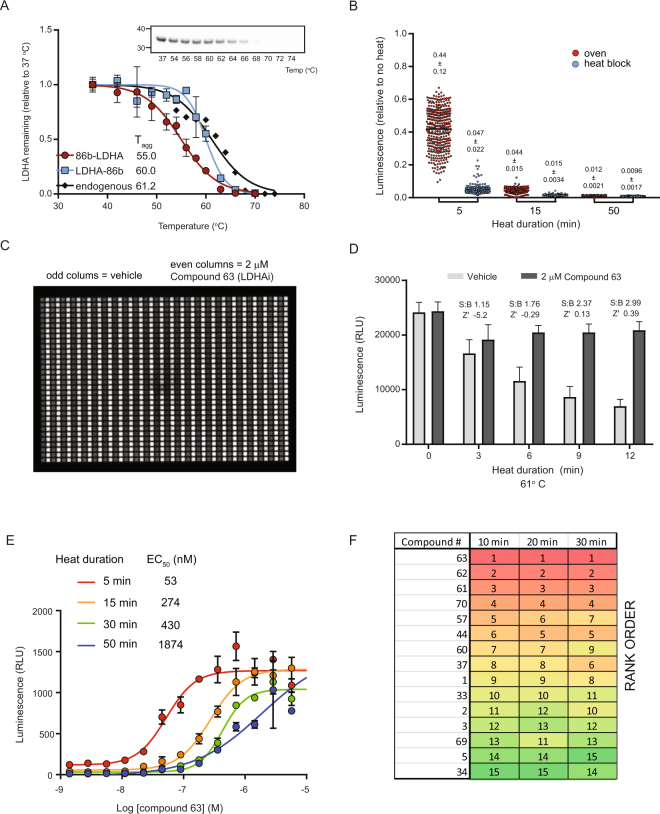
Optimization of a 1,536-well SplitLuc CETSA assay for qHTS. (**A**) Melt profiles of 86b-tagged and endogenous LDHA in HEK293T cells. Western blot for endogenous LDHA is shown in the inset. (**B**) LDHA-86b expressing cells were heated to 63.5 °C using convective (oven) or conductive (plate block) heat transfer (mean ± SD, n = 384). (**C**) LDHA-86b thermal stabilization was assessed by adding an LDHA inhibitor (Compound 63; 2 µM final concentration) to every other column of a 1,536-well plate. Samples were heated to 61 °C for 12 min and luminescence was measured by a ViewLux reader. (**D**) Assay statistics with LDHA-86b heated to 61 °C as a function of heat duration (mean ± SD, n = 768). Signal-to-background (S:B) indicates the fold change between groups treated with DMSO and compound. (**E**) Dose-dependent stabilization of LDHA-86b was assessed after heating samples to 63.5 °C for 5–50 min (mean ± SD, n = 2). AC_50_ values are right-shifted with increasing heat times. (**F**) Rank order of 15 LDHA inhibitors is maintained after heating 10, 20, or 30 min.

The SplitLuc CETSA assay was further miniaturized to a 1,536-well format for quantitative high-throughput screening (qHTS) using lactate dehydrogenase A (LDHA), a metabolic target for various cancers (Fig. 4A). Placement of the 86b fusion tag had an impact on the melt temperature, with the C-terminal fusion more closely matching the profile of endogenous LDHA (Fig. 4A). Similar to the IDH1(R132H) assay, freeze-thaw cycles and centrifugation steps were not required for the assay (Supplementary Fig. S4A,B). Use of a 1,536-well thermal cycler for the melting step posed challenges for automation and expansive screening, so other methods of heating were evaluated. Conductive heat transfer, using a custom machined aluminum plate block (Supplementary Fig. S4C), resulted in faster and more consistent LDHA melting compared to convective heating in an oven (Fig. 4B), providing a further simplification of the screening protocol. Favorable assay statistics for LDHA-86b stabilization using a recently characterized inhibitor, Compound 63^29^, were observed after 12 minutes of heating (Fig. 4C,D). Similar assay performance was observed for IDH1(R132H), although the optimal heat duration was 9 minutes (Supplementary Fig. S4D–F). As expected, inhibitor-mediated stabilization of LDHA-86b diminished with prolonged heating, with a right-shifting of AC_50_ values (Fig. 4E). The effect of heating time on compound activity was further evaluated by assessing a set of 15 LDHA inhibitor analogs (Supplementary Table S2)^29^. While increased heating times reduced the apparent compound potencies (Fig. 4E), the overall trend in rank order of compounds was maintained (Fig. 4F).

**Figure 5:**
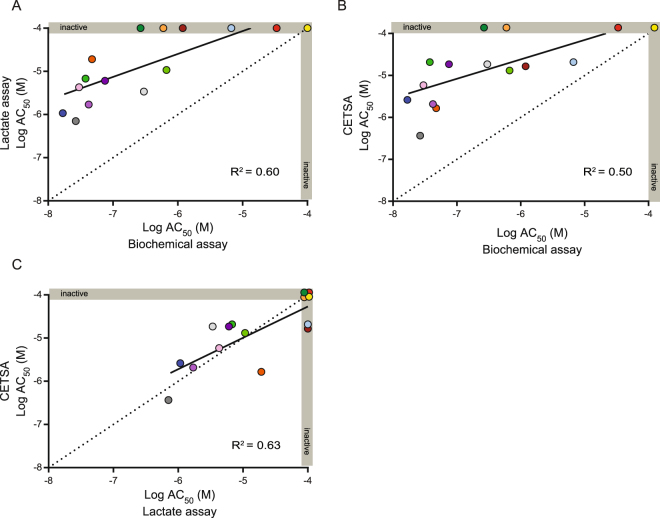
A comparison of LDHA inhibition among SplitLuc CETSA (63.5 °C for 7.5 min), biochemical, and cell-based assays. (**A**) Correlation plot of potencies for 15 LDHA inhibitors tested in a biochemical assay (x-axis) vs. a HEK293T cellular lactate production assay (y-axis). (**B**) Correlation plot of potencies for LDHA inhibitors in a biochemical assay (x-axis) vs. SplitLuc CETSA assay (y-axis). (**C**) Correlation plot of potencies for LDHA inhibitors in lactate production assay (x-axis) vs. SplitLuc CETSA complementation assay (y-axis). Greater agreement between AC_50_ values was observed for the two cell-based assays. Dotted line indicates equivalent potency in the two assays.

To compare the SplitLuc CETSA approach with an independent readout of functional activity, we continued to focus on LDHA, as decreased lactate secretion from cells can be measured upon its inhibition. A set of 15 inhibitors was tested in the following assays: (1) enzymatic activity using purified LDHA protein; (2) a cell-based lactate production assay; and (3) the SplitLuc CETSA. Shifts in potency were observed when the enzymatic assay was compared to lactate production (Fig. 5A and Supplementary Fig. S5A) or SplitLuc CETSA (Fig. 5B and Supplementary Fig. S5B), with loss of activity observed for several compounds in the cellular context. A stronger correlation was observed for the CETSA and lactate production assays, indicating better agreement for compound activities in the two cell-based assays (Fig. 5C and Supplementary Fig S5C).

**Figure 6:**
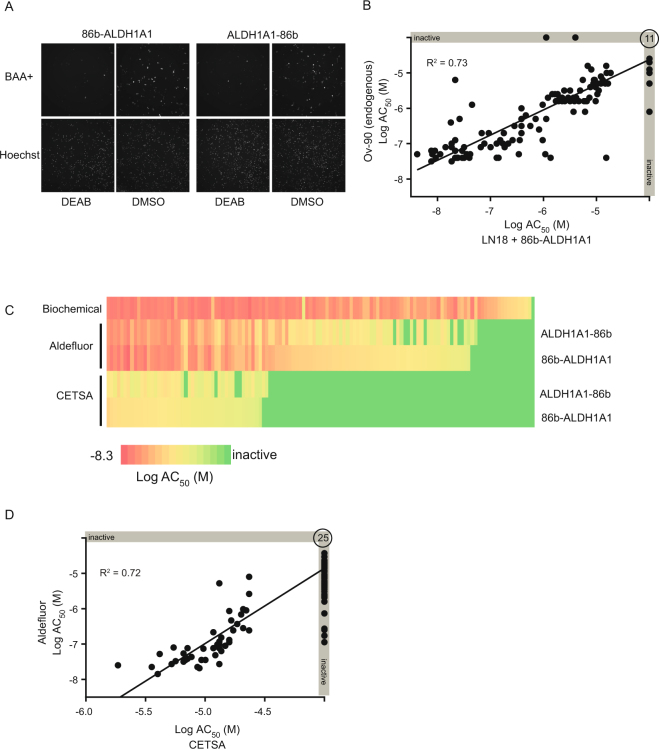
ALDH1A1 inhibitors (127 analogs) examined via the SplitLuc CETSA approach. (**A**) 86b-tagged ALDH1A1 retains cellular activity. LN18 cells were transfected with N- or C-tagged constructs and activities were examined using an Aldefluor high-content imaging assay that measures the conversion of BAAA (BODIPY-aminoacetaldehyde) to BAA (BODIPY-aminoacetate). Top images show BAA-retaining cells and bottom images show total cell number by nuclear staining with Hoechst. 4-N,N-diethylaminobenzaldehyde (DEAB) was used as a control inhibitor of ALDH1A1. (**B**) Inhibition of endogenous ALDH1A1 activity (OV-90 cells) is correlated with inhibition of 86b-ALDH1A1 as measured by Aldefluor assay. The correlation plot indicates compound’s LogAC_50_ in the Aldefluor assay in Ov-90 (y-axis) and 86b-ALDH1A1 transfected LN18 cells (x-axis). (**C**) Comparison of compound activities (LogAC_50_) measured with biochemical, Aldefluor (LN18), and SplitLuc CETSA (65  °C for 9 min) assay. The two cell-based assays were performed with 86b-tagged ALDH1A1 (N- and C-variants), while the biochemical assay utilized untagged protein. (**D**) The compound potencies from SplitLuc CETSA (65 °C for 9 min) and Aldefluor assays correlate, although potencies from the CETSA assay are right-shifted. Twenty-five compounds were inactive in both assays.

To explore the utility of the SplitLuc CETSA approach for assessing structure-activity relationships, we examined a set of aldehyde dehydrogenase 1A1 (ALDH1A1) inhibitors comprising 127 structurally-related analogs that were developed as part of an in-house medicinal chemistry campaign^30^. The 86b-tag did not abrogate functional activity of ALDH1A1 as both N- and C-tagged proteins were active in an imaging-based Aldeflour assay, which measures the oxidation of a fluorescent aldehyde substrate in LN18 cells that do not express endogenous ALDH1A1^31^ (Fig. 6A and Supplementary Fig. S6A). Moreover, the set of compounds inhibited the 86b-tagged ALDH1A1 in the Aldefluor assay, with good agreement for the N- and C-tagged variants (Supplementary Fig. S6B). Additionally, the set of compounds similarly inhibited 86b-tagged ALDH1A1 and endogenous ALDH1A1 proteins in OV-90 cells^30^, further supporting a minimal effect of the tag on the enzyme (Fig. 6B and Supplementary Fig. S6C). After determining T_agg_ and the optimal heating time (Supplementary Fig. S6D,E), thermal stabilization was examined for the N- and C-tagged proteins using the set of 127 compounds, and similar AC_50_ values were observed for the two variants (Supplementary Fig. S6F–H). The activity profile of the compounds was then directly compared across three assays: (1) an enzymatic assay using purified protein, (2) the cell-based functional Aldefluor assay, and (3) the SplitLuc CETSA. Whereas most compounds were highly active in the enzymatic assay, more variations in compound activity were observed among the cellular assays (Fig. 6C). Although there was a strong correlation between the two cell-based assays, potency was consistently right-shifted for the CETSA (Fig. 6D). Similar to IDH1(R132H), we tested the feasibility of running the SplitLuc assay for ALDH1A1 using cellular lysates. We observed thermal stabilization with the ALDH1A1 inhibitor NCT-505^30^, however, the potency and efficacy was lower compared to experiments performed in intact cells (Supplementary Fig. S6I). Moreover, thermal stabilization was only observed when lysates were supplemented with the ALDH1A1 cofactor NAD+ (Supplementary Fig. S6J).

**Figure 7:**
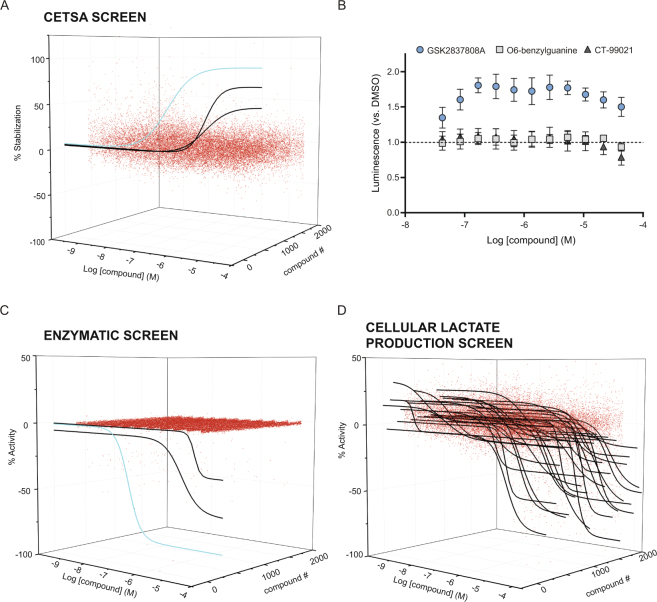
A primary screen of LDHA using the SplitLuc CETSA assay (63.5 °C for 9 min). (**A**) 1,850 samples from the mechanism interrogation plate (MIPE) library were screened in qHTS format as an 11-point concentration series for thermal stabilization of LDHA. The waterfall plot shows dose-response curves for three compounds classified as active in the screen. The response curve for GSK2837808A, a known LDHA inhibitor, is colored light blue. (**B**) Only the activity of GSK2837808A was confirmed when the three hits from the primary screen were re-tested (mean ± SD, n = 9). CETSA was performed at 63 °C for 3.5 min in 384-well PCR plates (thermal cycler). (**C**) MIPE compounds were screened for activity using an LDHA enzymatic assay. Three actives, including GSK2837808A (light blue curve), were identified. The remaining two actives did not overlap with the compounds identified in the CETSA assay. (**D**) MIPE compounds were screened for activity with the lactate production assay. Three-hundred and seventy-six compounds (20.3% hit rate) were identified as active (black curves).

**Figure 8:**
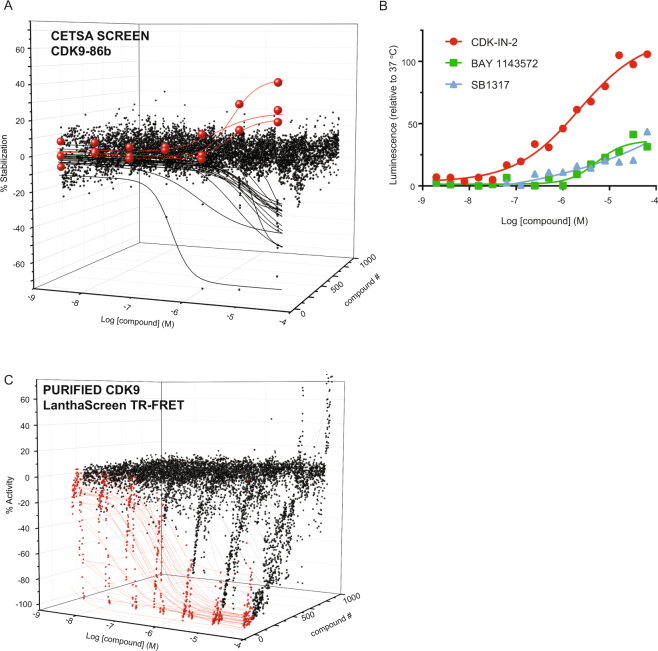
A primary screen of CDK9 using the SplitLuc CETSA assay. (**A**) A collection of 977 kinase inhibitors were screened in qHTS format as a 7-point concentration series for thermal stabilization of CDK9 (45 °C for 9 min, 1536-well plates). The waterfall plot shows dose-response curves for three compounds (red) classified as active in the screen. Dose-response curves of “destabilizers” are shown in black. (**B**) The stabilizing activities of all 3 CDK9 inhibitors found in the primary screen were confirmed in follow-up experiments (45 °C for 3.5 min, 96-well PCR plate, thermal cycler). (**C**) The kinase inhibitor collection was screened for activity using a Lanthascreen binding assay with purified CDK9/cyclin K. Eighty-nine hits (red curves) with submicromolar activity were identified.

Finally, we examined the feasibility of utilizing SplitLuc CETSA for a primary screen of two targets, LDHA and cyclin dependent kinase 9 (CDK9). First, we performed a qHTS screen of 86b-tagged LDHA against a collection of 1,850 oncology-focused and mechanistically annotated compounds (Mechanism Interrogation PlatE)^32^ with each compound tested as an 11-point concentration series. Three compounds were identified as active, and the most potent and efficacious stabilization was observed for GSK2837808A, the only characterized LDHA inhibitor in the library (Fig. 7A and Supplementary Fig. S7A). Follow-up experiments using SplitLuc CETSA confirmed the other two hits were false positives, as only GSK2837808A confirmed (Fig. 7B). A biochemical screen for LDHA enzymatic activity also identified three actives from the library, with the only common hit being GSK2837808A (Fig. 7C and Supplementary Fig. S7B). The low hit rate observed is in agreement with previous screening efforts for this target^29^. In contrast to CETSA, tracking lactate production as a cell-based readout of LDHA activity identified 376 compounds as active (Fig. 7D and Supplementary Fig. S7C). Hence, for this particular metabolic target where the phenotypic loss-of-signal cellular assay is prone to the identification of off-target activities, CETSA provides an alternative cell-based screening platform with an outcome similar to that of isolated target. Second, we performed a qHTS screen of CDK9 against a collection of 977 kinase inhibitors composed of both clinical and pre-clinical-stage compounds. Prior to screening, thermal stabilization of CDK9-86b was validated using LY2857785 and the assay was optimized in 1536-well format (Supplementary Fig. S8A–C). From the screen, three compounds with annotated CDK9 inhibitory activity showed thermal stabilization of CDK9-86b (Fig. 8A). Follow-up experiments indicated that all three compounds, CDK-IN-2, BAY 1143572 and SB1317, stabilize CDK9-86b, with CDK-IN-2 being the most potent and efficacious (Fig. 8B). Interestingly, the screen also identified compounds that decreased luminescence signal (Fig. 8A). A counterscreen using parental HEK293T cells indicated that the majority of these compounds constitute artifacts that either destabilize the complementation between NanoLuc fragments or inhibit NanoLuc activity (Supplementary Fig. S8D). We then tested the kinase inhibitor collection against a Lanthascreen-based binding assay using purified CDK9-cyclin K. In contrast to the low hit rate in SplitLuc CETSA, 89 compounds (9%) showed submicromolar activity in the binding assay using purified protein (Fig. 8C and Supplementary Table S3).

## Discussion

Reliable cell-based assays are crucial for the successful development of small molecules that modulate biological processes. Cellular target engagement studies, using CETSA, provide a direct readout of compound binding to a protein of interest in an intact cellular environment, and confidence in on-target activity. Here, we have developed, optimized and validated a SplitLuc CETSA approach to facilitate target engagement studies that can be implemented in 96-, 384-, or 1,536-well formats. The broad applicability of the assay was successfully demonstrated for a diverse set of targets that span multiple functions, sizes, structures, and subcellular localizations. The assay was validated using several complementary cell-based assays, which allowed comparisons of target engagement with orthogonal readouts of functional activity. The simplicity of the SplitLuc CETSA approach allows rapid implementation and has the potential to provide insight into intracellular target engagement, particularly when other cell-based assays are not available, have insufficient throughput, or are sensitive to a wide variety of off-target mechanisms (such as metabolic phenotypic assays).

Not all proteins are expected to demonstrate a thermal shift with compound binding. Furthermore, the magnitude of thermal shift is expected to vary widely for different proteins, and targets that lack thermal shift or show small changes in thermal stability will not be amenable to CETSA screening. Additionally, not all compounds that modulate a target will induce a thermal shift, and these compounds would be missed in a CETSA screen. While targets need to be assessed on an individual basis, there are several additional aspects of the SplitLuc CETSA that should be considered during implementation. First, the 86b tag might alter thermal stability, enzymatic activity, subcellular localization, or protein interactions. While it is conceivable that the peptide tag itself could alter thermal stability, the 86b-tag did not show an obvious effect on the apparent melting temperature of 18 fusions when compared to untagged endogenous proteins. Similarly, aggregation of 86b-tagged SOD1, a thermostable protein often used as a loading control for CETSA, was not observed, consistent with the peptide tag having minimal contribution to T_agg_ (data not shown). No deleterious effects of 86b on activity were observed for mutant IDH1 or ALDH1A1, but it is important to confirm expected activity for each new construct when the target has a known activity and an available functional assay. Secondly, when using transient transfections, the expression levels may not match endogenous levels in the cell type under investigation. As such, it is important to consider that absolute expression levels of a target can impact apparent compound potency, particularly when comparing between cell types or independent cell-based assays. In circumstances where precise control of expression is desired, transfection conditions can be optimized to approximate endogenous expression. Alternatively, 86b can be introduced into the endogenous locus using genomic engineering technologies (*e.g*. CRISPR/Cas9), a procedure that is simplified by the small size of the peptide tag^33^. While the majority of our experiments were performed with HEK293T cells, the choice of cell line may impact the results. For IDH1(R132H), thermostabilization was similar among the four cell lines tested (Supplementary Fig. S1H), but we anticipate that some proteins will exhibit different responses across cell types. This consideration may be particularly pertinent for proteins with tissue-restricted expression profiles, where the appropriate protein-protein interactions (interactome) may be required for informative CETSA.

Recently, a high-affinity NanoLuc complementation system based on the luminescent NanoBiT enzyme was commercialized by Promega (HiBiT)^22^. The HiBiT technology also utilizes the peptide 86, which is detected using a proprietary complementation substrate. Although we did not test the HiBiT system directly, we anticipate that HiBiT reagents will be well-suited for high-throughput CETSA. A CETSA assay platform based on the DiscoverX β-galactosidase enzyme fragment complementation technology was also recently reported by GlaxoSmithKline^21^. With this approach, target proteins are fused to the DiscoverX 42-amino acid ePL tag at the C-terminus and expressed in cells via BacMam transduction. Focusing on SMYD3 and IDO1 targets, the authors show that the ePL tag does not change target T_agg_ nor the magnitude of compound-mediated stabilization and that centrifugation and freeze-thaw cycles of traditional CETSA can be omitted altogether to successfully perform assays in 384-well format, which is in agreement with our findings.

We observed consistency in T_agg_ when transitioning between plate formats using a thermal cycler, but each target required optimization when miniaturizing to a 1,536-well format using conductive heat transfer. In all formats, the T_agg_ and duration of heat transfer will have an impact on SplitLuc CETSA results. For instance, potency values are right-shifted when the heat duration is increased, as demonstrated for LDHA. A rightward shift in compound potency was also reported for a set of SMYD3 inhibitors when CETSA and cellular methylation assays were compared^21^. In the context of high-throughput screening, prolonged heating times are expected to reduce the capacity to detect compounds that bind weakly, which highlights the importance of empirically defining the optimal assay conditions for each target, while also taking into consideration the acceptable hit rate for the screening campaign.

Trypan Blue exclusion studies using a set of cell lines from different tissue origins suggest that experiments should be interpreted with caution when heating above ~55 °C, as membrane integrity can be compromised. Notably, Trypan Blue may enter cells differently than other small molecules, so these results are only an approximation of membrane integrity. For assays performed at temperatures above 55 °C, it is worth considering a wash step to remove extracellular compound before heating. This step is expected to minimize target engagement by extracellular compound if the membrane integrity is compromised during heating. From a screening perspective, we propose performing initial experiments using a no-wash method, regardless of the melt temperature. Hits can then be confirmed using washes in follow-up experiments where medium exchanges are easier to implement. Additionally, assays that are better suited to directly evaluate membrane permeability (*e.g*. direct quantitation of intracellular concentration by mass spectrometry^34^) can be implemented for compounds identified in CETSA at temperatures above 55 °C.

CETSA can also be performed by adding small molecules directly to cellular lysates, which removes the membrane permeability component of the assay, but can provide useful information about binding in a complex protein environment. However, lysis can also disrupt subcellular compartmentalization and molecular concentrations (including proteins, cofactors, *etc*.), which may impact the results. For example, our studies of ALDH1A1 demonstrate that its cofactor, NAD+, must be added to lysates to maintain NCT-505 engagement. In concept, assays performed at elevated temperatures, where membranes are disrupted, may behave similarly to those performed in lysates, although this will be dependent on the compounds and target under investigation.

We demonstrated the ability of the SplitLuc CETSA assay to guide SAR studies. By comparing the activity of a set of 127 structurally-related ALDH1A1 inhibitors in cell-based Aldefluor and CETSA assays, we showed a good correlation between assays; however, the apparent compound potencies from the CETSA assay were weaker (right-shifted) compared to those of the Aldefluor assay. This shift in potency might result from the stringency of melt conditions, the non-equivalence for enzymatic activity and thermostabililzation (both of which are impacted by binding kinetics), and/or inherent assay sensitivity for this target. Nevertheless, the SplitLuc CETSA assay allowed us to identify a set of leads where the observed inhibitory activity on the Aldefluor assay likely indicates on-target specificity within a cellular context. This suggests that the SplitLuc CETSA assay can be considered for guiding SAR, particularly for discovery programs where alternative cell-based assays are unreliable or unavailable.

The unprecedented ability to miniaturize a CETSA assay to a 1,536-well format allowed us to perform a primary qHTS to identify compounds that thermostabilize LDHA and CDK9. For LDHA, of 1,850 compounds tested, only one compound stabilized the protein at 63.5 °C. The low hit rate for LDHA engagement is in agreement with previous large-scale screening efforts, where only one viable chemotype emerged from a 400,000-compound screen^29,35^. Additionally, the low hit rate for the SplitLuc CETSA suggests the assay is not prone to spurious false positives due to non-specific compound binding/stabilization or effects on NanoLuc complementation. Comparison with the cell-based lactate production assay, which identified 376 compounds that decreased lactate production, highlights the off-target activities that can impinge on a phenotypic assay. Similar to the LDHA scenario, the SplitLuc CETSA assay identified only three CDK9-86b stabilizers, which have demonstrated CDK9 inhibitory activity. A cell-free CDK9 binding assay had a much higher hit rate, with ~9% of the kinase inhibitors showing potent (submicromolar) CDK9 binding capabilities. The kinase inhibitor collection also contained numerous compounds that either destabilize complementation or inhibit NanoLuc activity, as revealed by a counterscreen using 86b peptide.

We anticipate that the method presented here will be useful for the study of chemical chaperones, as highlighted by studies of CFTR and glucocerebrosidase. Similar approaches can be employed for pharmacological chaperone screens, including for rare missense mutations where other cell-based assays are not available or would require significant resources to develop. Additionally, it is conceivable that the SplitLuc CETSA assay could be used to identify small molecule binders of a protein target of interest to pursue target degradation strategies, by coupling the identified small molecule with a protein degradation warhead (*e.g*. PROTAC). A more complete automation of the 1536-well SplitLuc assay, by integrating the hardware onto a robotic screening platform, will be the focus of future work. We foresee it will allow the implementation of CETSA as a primary high-throughput screening assay, in some instances replacing assays using isolated proteins altogether. In summary, we present a homogeneous, target-independent CETSA platform compatible with both 384- and 1,536-well plate formats, which has the potential to be broadly adopted by the scientific community and catalyze drug discovery and probe development.

## Experimental Procedures

### Compounds

ALDH1A1 inhibitors have been recently described^30^. LDHA inhibitors were previously described in Rai *et al*.^29^. IDH1 inhibitors were previously described in Urban *et al*. and Davis *et al*.^24,36^. Other compounds used in the study were Methotrexate (MedChem Express, HY-14519), Panobinostat (Selleck Chem, S1030), AG-221 (MedChem Express HY-18690), Isofagomine (Toronto Research Chemicals #I816010), Lumacaftor (BioVision #2857), and LY2857785 (Medchem Express, HY12293). For qHTS, compounds in the mechanism interrogation plate (MIPE) were tested in 11-points (intraplate, 1:3 dilution factor). The kinase inhibitor collection containing 977 kinase inhibitors was tested in 7-point doses (interplate, 1:5 dilution factor). In all cases, DMSO was used as vehicle.

### Cloning

pcDNA3.1-based vectors were created for N- and C-terminal tagging by linearizing pcDNA3.1(+) with NheI and EcoRI and inserting the peptide tag using an oligonucleotide duplex and In-Fusion reagents (Clontech). For the C-terminal tag, the duplex was: Sense: 5′-ACCCAAGCTGGCTAGCAACTAACGGATCCGTGAGTGGCTGGCGACTGTTCAAGAAGATCAGCGGCAGCTAA GGCGCGCCGAATTCTGCAGATAT-3′ and Antisense: 5′- ATATCTGCAGAATTCGGCGCGCCTTAGCTGCCGCTGATCTTCTTGAACAGTCGCCAGCCACTCACGGATCCgttagttGCTAGCCAGCTTGGGT-3′. Notably, the first amino acids of the peptide tag (Gly-Ser) were encoded using GGATCC, which is a BamHI site and facilitated downstream cloning steps. For the N-terminal fusion tag, the duplex was Sense: 5′-ACCCAAGCTGGCTAGCCACCATGGGCAGCGTGAGTGGCTGGCGACTGTTCAAGAAGATCAGCGGATCCAACTAACAATAGCGTTATCGAATTCTGCAGATATC-3′ and Antisense: 5′- GATATCTGCAGAATTCGATAACGCTATTGTTAGTTGGATCCGCTGATCTTCTTGAACAGTCGCCAGCCACTCACGCTGCCCATGGTGGCTAGCCAGCTTGGGT-3′. In this case, a BamHI site encodes the final two amino acids of the peptide tag. Open Reading Frames (ORFs) of the genes of interest were cloned into the acceptor backbones using BamHI/EcoRI (N-terminal) or NheI/BamHI (C-terminal) sites by PCR amplifying the coding region with InFusion compatible oligonucleotides, defined in detail in Supplementary Table S1. IDH1, LDHA, DHFR, GC, and ALDH1A1 constructs were prepared by GeneArt Gene synthesis (ThermoFisher) in pcDNA3.1 (+). For Gateway cloning (IDH2 constructs only), a destination vector containing the 86b tag was created by replacing the V5 tag in pcDNA3.2-V5/DEST. The plasmid was digested with PmeI and SacII and an oligo duplex inserted by InFusion. The oligo was: Sense: 5′- TGATCTAGAGGGCCCGCGGGGCAGCGTGAGTGGCTGGCGACTGTTCAAGAAGATCAGCGGCAGCTAAGTTTAAACGGGGGAGGCTA-3′ and Antisense: TAGCCTCCCCCGTTTAAACTTAGCTGCCGCTGATCTTCTTGAACAGTCGCCAGCCACTCACGCTGCCCCGCGGGCCCTCTAGATCA-3′. The Gateway strategy leaves an additional C-terminal ‘scar’ after the final amino acid of the ORF, which encodes “LPTFLYKVVDLEGPR” prior to the 86b tag.

### Cell culture

HEK293T, LN18, OV-90, HeLa, 22RV1, MDA-MB-468, and HT-29 cells were obtained from ATCC (CRL-1573, CRL-2610, CRL-11732, CCL2, CRL-2505, HTB-132, and HTB-38, respectively). HuCC-T1 and CC-LP-1 were a kind gift of Dr. N. Bardeesy (Massachusetts General Hospital, Boston). HEK293T cells were cultured in DMEM (4.5 g/L glucose) with 10% fetal bovine serum (FBS), 6 mM L-glutamine, 1 mM sodium pyruvate, 50 U/mL penicillin, and 50 µg/mL streptomycin. LN18, HT-29, CC-LP-1, and HeLa cells were cultured in the above medium, without sodium pyruvate. OV-90 cells were cultured in a 1:1 mixture of MCDB 105 medium (Cell Applications Inc.) and M199 medium (HyClone, GE), supplemented with 15% FBS, 1% Penicillin-Streptomycin (Life Technologies), and a final concentration of 1.85 g/L sodium bicarbonate (HyClone, GE). 22RV1, HuCC-T1, and MDA-MB-468 cells were grown in RPMI 1640 supplemented with 10% FBS, 6 mM L-glutamine, 100 units/mL penicillin, 100 µg/mL streptomycin. All cells were grown at 37 °C in a humidified incubator maintained at 5% CO_2_ and tested negative for mycoplasma using a MycoAlert detection kit (Lonza).

### Recombinant protein and peptide production and purification

 11S and 86b proteins were produced by GenScript. For recombinant 11S fragment, the coding DNA sequence was fused to a 6x His tag to facilitate downstream purification and the sequence was subcloned into an *E. coli* expression vector. BL21 Star (DE3) cells were transformed with recombinant plasmids and a single colony was inoculated into LB medium containing IPTG for induction of protein expression. SDS-PAGE and Western blot analysis were used to monitor the expression and select optimal harvest timing. Cells were harvested by centrifugation and pellets were lysed by sonication. After His-tag purification, fractions were sterilized through a 0.22 μm filter. Proteins were analyzed by SDS-PAGE and Western blot using standard protocols and a mouse-anti-His mAb (GenScript, Cat.No.A00186). The concentration of purified protein was determined using a Bradford protein assay with BSA as a standard. Protein was stored in 1X PBS, 10% glycerol, pH 7.4, and purity was approximately 90%, as estimated by densitometric analysis of the Coomassie Blue-stained SDS-PAGE gel under reducing conditions. The 15 amino acid 86b peptide was stored in ultrapure dH_2_O and was 99.9% pure by HPLC.

### Low-throughput (96-well PCR plates or strips) CETSA complementation assay

Cells were transfected in 6-well dishes using a reverse transfection procedure, where 1.25 ml of complexes (6.25 µL Lipofectamine 2000 and 3 µg DNA per well) was combined with 1.25 ml of HEK293T cell suspension (1 × 10^6^/mL, 1.25 × 10^6^ cells total). For CDK9, 2 µg of DNA per well was used. After 24 h (48 h for CDK9), cells were harvested by trypsinization and resuspended at 1 × 10^6^ cells/mL (unless otherwise indicated) in CETSA buffer containing DPBS (with CaCl_2_ and MgCl_2_) plus 1 g/L glucose, 1X Halt protease inhibitor cocktail (ThermoFisher), and 0.5% DMSO (DMSO was not added for experiments that received subsequent compound and vehicle treatment). For temperature range experiments (without compound or single dose), samples were aliquoted to PCR strips at 30 µL per tube. Compound was added and cells were incubated at 37 °C for 1 h. Samples were then heated for 3.5 min using a pre-heated thermal cycler, allowed to equilibrate to room temperature, and 6 µL of 6% NP40 was added to each well. For freeze-thaw experiments, tubes were placed in an aluminum PCR block on a dry ice/ethanol bath for 3 min followed by incubating at 37 °C for 3 min, vortexing for 3 sec, and repeating these steps three additional times. 11S (GenScript) and furimazine substrate (from Nano-Glo Luciferase Assay System, Promega) were added, at final concentrations of 100 nM and 0.5X, respectively, and samples were analyzed for luminescence intensity using a ViewLux High-throughput CCD imager (Perkin Elmer) equipped with clear filters.

### 384-well CETSA assay

Cells were transfected in T75 flasks using a reverse transfection procedure, where 9 mL of complexes (45 µL Lipofectamine 2000 and 22.5 µg DNA) was combined with 10 mL of HEK293T cell suspension (1 × 10^6^ cells/mL, 10 million cells total). After 24 h, cells were harvested by trypsinization, resuspended at 1 × 10^6^ cells/ml as described above and dispensed (15 µL cells/well) into 384-well PCR plates (Roche) using a Multidrop Combi (ThermoFisher). Compounds (63 nL) or DMSO vehicle control (63 nL) were subsequently pinned using a pin tool (GNF) and incubated for 1 h at 37 °C. Plates were sealed and heated at the indicated temperature for 3.5 min and cooled to 25 °C using AB qPCR machine (Roche) using ramp speed of 1.5 °C/sec for heating phase and max ramp rate for the cooling phase. Three µL of 6% NP40 were added per well and incubated for 30 min to allow cell lysis followed by addition of 11S and furimazine substrate (at final concentrations of 100 nM and 0.5X, respectively). Samples were analyzed for luminescence intensity using a ViewLux reader.

### 1,536-well CETSA assay

Cells were transfected and harvested as above (384-well protocol). Cells were dispensed (5 µL cell/well) into 1,536-well white plates (Aurora, cyclic olefin polymer, cat# EWB041000A) using a Multidrop Combi. Compounds (23 nL) were subsequently pinned using a pin tool (Wako Automation) and incubated for 1 h at 37 °C. Plates were heated at the indicated temperature and time using a heating block (see below) and cooled to 25 °C. One µL of 6% NP40 was added per well and plates were incubated for 30 min to allow cell lysis followed by addition of 3 µL 11S (final concentration 100 nM) and furimazine substrate. Plates were centrifuged and analyzed for luminescence intensity using a ViewLux reader. For the LDHA and CDK9 screens, a final concentration of 0.5X and 0.25X furimazine was used, respectively. Normalization controls include DMSO and GSK2837808A-treated samples or unheated samples for LDHA and CDK9, respectively. The CDK9 counterscreen was performed as above with the following differences: 5 µL of untransfected HEK293T cells (1 × 10^6^/mL in CETSA buffer) were dispensed into 1,536-well plates followed by compound addition, 37 °C incubation, heating and lysis steps as above. Subsequently, 500 pM (final) of 86b was added to the wells, followed by addition of 100 nM 11S and 0.25X furimazine substrate (final).

The aluminum block for heating a 1,536-well plate was designed by measuring a plate to determine the dimensions of the area bordered by molded-in reinforcing ribs in X and Y, and the bottom well face and bottom flange face in Z. Then a block to be machined from 6061 T6 aluminum plate was molded, which would be a free fit with approximately 0.5 mm clearance for the plate in all axes. The block was machined using a manual vertical milling machine, end mills and a face mill. For oven heating experiments, plates were placed in middle rack of convection lab oven (ThermoFisher) and covered with metal lids to minimize evaporation.

### CETSA in cellular lysates

Cells were collected in CETSA buffer at 1.0 × 10^6^ cells/mL (as outlined above) and NP40 was added to a final concentration of 0.4%. After rotating samples end-over-end for 30 min (room temperature), lysates were clarified by centrifugation at 20,000 × g for 10 min (4 ^o^C). Cofactor (*e.g*. NAD+) was added to the clarified lysate. For temperature response experiments, 25 µL of lysate was transferred to PCR tubes and heated for 3.5 min to various temperatures. For isothermal dose-response experiments, 5 µL of clarified lysate was dispensed to 1536-well plates using a BioRAPTR FRD workstation and samples were heated on an aluminum block. Samples were allowed to equilibrate to room temperature and substrate was added (final concentration: 100 nM 11S, 0.5X furimazine). Luminescence was captured using a ViewLux imager as outlined above.

### Western blots

Samples were processed as outlined in low-throughput complementation assay section, using freeze-thaw cycles for lysis. Lysates were centrifuged at 15,200 × g for 20 min at 4 °C. Samples were run on a 4–12% Bis-Tris NuPAGE gel (ThermoFisher) using MOPS buffer and transferred to a PVDF membrane using an iBlot 2 transfer system at 25 V for 7 min. Membranes were blocked with a 5% milk solution (50 mM Tris HCl, pH6; 150 mM NaCl; 0.1% Tween-20) and primary antibodies were incubated overnight at 4 °C in blocking solution. Antibodies were: anti-IDH1 ([D2H1], Cell Signaling #8137, 1:500), anti-IDH1(R132H) (Millipore #MABC171, 1:500), anti-LDHA ([C4B5], Cell Signaling #3582, 1:1000). Anti-rabbit/mouse-HRP (rabbit = Cell Signaling 7074; mouse = Cell Signaling # 7076) was added at 1:2500 and incubated for 1 h at room temperature. A chemiluminescent signal was generated with Supersignal west dura solution (ThermoFisher) and captured using a Biorad Chemidoc system. Densitometric analysis was performed using Photoshop software (Adobe).

### 2-HG assay

HEK293T cells reverse transfected in 24 well dishes by adding 2.5 × 10^5^ cells onto transfection complexes (0.75 µg plasmid DNA, 1.5 µL Lipofectamine 2000 per well). Cell culture medium was collected after 72 h and 15 µL was transferred to 384-well opaque plate. 1.5 µL of 660 mM HCl was added to each well and samples were incubated at room temperature for 10 min. 1.5 µL of 720 mM Tris-HCl base was added to each well. Then, 52 µL of 2-HG detection solution (100 mM HEPES (pH 8.0), 100 µM NAD + , 1 µg/mL active recombinant D-2-Hydroxyglutarate dehydrogenase (D2HGDH; Biovision, Cat: P1001), 5 µM resazurin, 0.03 mg/mL diaphorase (Sigma, Cat: D5540) was added and the plate was incubated at room temperature. A 2-HG standard curve was prepared in 100 mM HEPES (pH 8.0). Fluorescence was measured on a ViewLux reader using Ex: 525, Em: 598/25 filters.

### Biochemical assays

The LDHA biochemical assay was performed as previously described^29^. Briefly, 3 µL of human lactate dehydrogenase 5 (2 nM final) (no. A38558H, Meridian Life Science, Inc.) in LDH assay buffer (200 mM Tris HCl, pH 7.4, 100 µM EDTA, and 0.01% Tween-20) was added to a black solid bottom 1536-well assay plate (Greiner Bio-One). Following compound pin transfer (23 nL), 1 µL of substrate solution containing NADH (0.06 mM final) and sodium pyruvate (0.2 mM final) (Sigma-Aldrich) in LDH assay buffer was dispensed to initiate the reaction. After 5 min incubation at room temperature, 1 µL of detection reagent [C. kluyveri diaphorase (0.133 mg/mL final, Sigma-Aldrich) and resazurin sodium salt (37 µM final, Sigma-Aldrich) in LDH assay buffer] was added to each well. Plates were immediately transferred to a ViewLux reader and any resulting resorufin fluorescence was measured (ex 540 nm, em 590 nm) at 0 and 10 min. Fluorescence was normalized using enzyme-free and DMSO-treated control wells on each plate.

The ALDH1A1 biochemical assay using untagged protein was performed as previously described^31,37^. Briefly, 3 µL of 20 nM purified human ALDH1A1 in assay buffer (100 mM HEPES pH 7.5 with 0.01% Tween 20) were dispensed into a 1,536-well solid-bottom black plate (Greiner Bio One). Twenty-three nL of compounds or control Bay 11–7085 were transferred via pin tool (Wako Automation). Samples were incubated (room temperature, protected from light) for 15 min followed by a 1 µL substrate addition of NAD + and Propionaldehyde (final concentrations of 1 mM and 80 µM, respectively). Plates were centrifuged and read in kinetic mode on a ViewLux imager equipped with 340 nm excitation, 450 nm emission filters for 5 min. The change in fluorescence intensity over the 5 min was normalized using enzyme-free and DMSO-treated control wells on each plate.

The TR-FRET Lanthascreen Eu Kinase Binding assay for CDK9/cyclin K was purchased from ThermoFisher and performed as per manufacturer instructions. Briefly, 4 µL of a master mix containing 4 nM CDK9/Cyclin K (Invitrogen #PV4335), 2 nM Biotin Anti-His Ab, 2 nM Eu-Streptavidin, and 10 nM Kinase Tracer 236 Solution in 1X Kinase Buffer were dispensed into Greiner 1,536-well white medium binding plates using a BioRAPTR FRD workstation. Twenty-three nL of compound and controls (DMSO and LY2857785 at a final concentration of 6 µM) were immediately delivered to the assay plates via pin tool transfer. The plates were allowed to incubate for 1 h at room temperature, and TR-FRET fluorescence was subsequently measured with a PerkinElmer EnVision Multilabel plate reader (Ex: 317/20; Em 1: 620/12; Em 2: 665/12; Lag time 100 µs; Integration time 200 µs).

### Lactate production assay

HEK293T cells were cultured as described above. Cells were rinsed with PBS, trypsinized, and resuspended in phenol red free DMEM (Life Technologies) without supplements. Cells were immediately plated to 1536-well black clear bottom plates (Corning) at 250 cells per well in 4 µL volume. Compound or vehicle control was added to wells via pin tool transfer and cells were incubated at 37 °C for 1 h. Two µL of lactate reaction mixture (Biovision K607-100) was added to each well and plates were covered and incubated at room temperature for 30 min. Fluorescence was measured using a ViewLux microplate imager equipped with Ex/Em 528/598 nm filters.

### Aldefluor assay

For initial determination of 86b-tagged ALDH1A1 activity, cells were transfected using a reverse transfection procedure, where 1 mL of complexes (6 µL Lipofectamine 2000 and 3 µg DNA) was combined with 1 mL of LN18 cell suspension (5 × 10^5^/mL) and 100 µL/well of mix was dispensed into black, clear-bottom 96-well plates (Corning). After 24 h, media was removed and replaced with 100 µL/well of Aldefluor buffer (STEMCELL Technologies) containing BAAA substrate and Hoechst 33342 (ThermoFisher, final concentrations of 500 nM and 0.5 nM, respectively). Vehicle DMSO or DEAB (1 µM final) was subsequently added and plates were incubated for 30 min at 37 °C to allow the conversion of BAAA into BAA. Cells were washed and 100 µL/well of Aldefluor buffer was dispensed before imaging on an IN Cell 2200 (GE Healthcare). For high-throughput assays, LN18 cells were transfected in T75 flasks using a reverse transfection procedure as described above for high-throughput HEK293T CETSA assays. After 16 h, cells were harvested and plated (1,000 cells/well/5 µL) into black, optical quality clear bottom, TC treated 1,536-well plates (Aurora Microplates) using a Multidrop Combi dispenser and incubated overnight at 37 °C. The high-content Aldefluor assay was subsequently performed on transfected LN18 cells or untransfected OV-90 as previously described^31^. Images were analyzed using the IN Cell Investigator v1.6.2 analysis software’s canned Multi-Target Analysis algorithm (GE Healthcare) as described.

### Membrane thermal integrity assay

30,000 HEK293T cells were prepared in 30 μL CETSA buffer containing 1X protease inhibitor cocktail (ThermoFisher) supplemented with 0.5% DMSO (1.5% DMSO total). For the DMSO tolerance experiment, DMSO was added to DPBS at 0, 1, 2, or 3%. Cell suspensions were heated for 3.5 min at 42 to 74 °C, using 4 degree intervals, then removed to an aluminum block on wet ice. Cell suspensions were mixed with equal parts Trypan Blue (LONZA) ([final] = 0.2% trypan blue) and counted immediately using a C-chip hemocytometer (iNCyto, Korea). Trypan positive (permeabilized) and negative (intact) were counted, with n = 2 at each temperature. For additional cell lines, one million cells were prepared in 100 µL of phenol-red free DMEM containing 1% DMSO. Cells were heated for 3 min over 42 to 90 °C at 4 degree intervals, then removed to an aluminum block on wet ice. Membrane integrity was assessed as described above.

### PAMPA

The stirring double-sink PAMPA method patented by pION Inc. (Billerica, MA) was used to measure compound permeability as previously described^38^. Compounds were diluted in donor and acceptor solutions buffered to pH7.4 and DMSO concentration was 0.5%. Permeability calculations were performed using Pion Inc. software.

### qHTS analysis

Data from each assay were normalized plate-wise to corresponding controls as described previously^39^. The same controls were also used for the calculation of the Z’ factor for each assay. Percent activity was derived using in‐house software (http://tripod.nih.gov/curvefit/). All concentration–response curves were fitted and AC_50_ were calculated with the GraphPad Prism software; curves were classified as described previously^27^. Area under the curve (AUC) was calculated using the trapezoidal rule to approximate area between the response curve and the x-axis across the concentration range. Equivalent concentration ranges were used for all compounds within an experiment. Efficacy cutoff of 3 σ from the mean (of vehicle control) was used to classify active compounds.

## Electronic supplementary material

Supplementary Figures S1-S8 and Tables S1-S3
