# Supplementary material for: A widely-applicable high-throughput cellular thermal shift assay (CETSA) using split Nano Luciferase
Source: Sci Rep. 2018 Jun 21;8:9472. doi: 10.1038/s41598-018-27834-y (PMC6013488; doi:10.1038/s41598-018-27834-y)
Supplement: Supplementary file 1 — Supplementary Figures S1-S8 and Tables S1-S3 [file 41598_2018_27834_MOESM1_ESM.pdf]

SUPPLEMENTARY INFORMATION

A widely-applicable high-throughput cellular thermal shift assay (CETSA) using split Nano Luciferase.

Natalia J Martinez, Rosita Asawa, Matthew G Cyr, Alexey Zakharov, Daniel J Urban, Jacob Roth, Eric Wallgren, Carleen Klumpp-Thomas, Nathan P Coussens, Ganesha Rai, Shyh-Ming Yang, Matthew D Hall, Juan J Marugan, Anton Simeonov, Mark J Henderson

Supplementary Figure 1

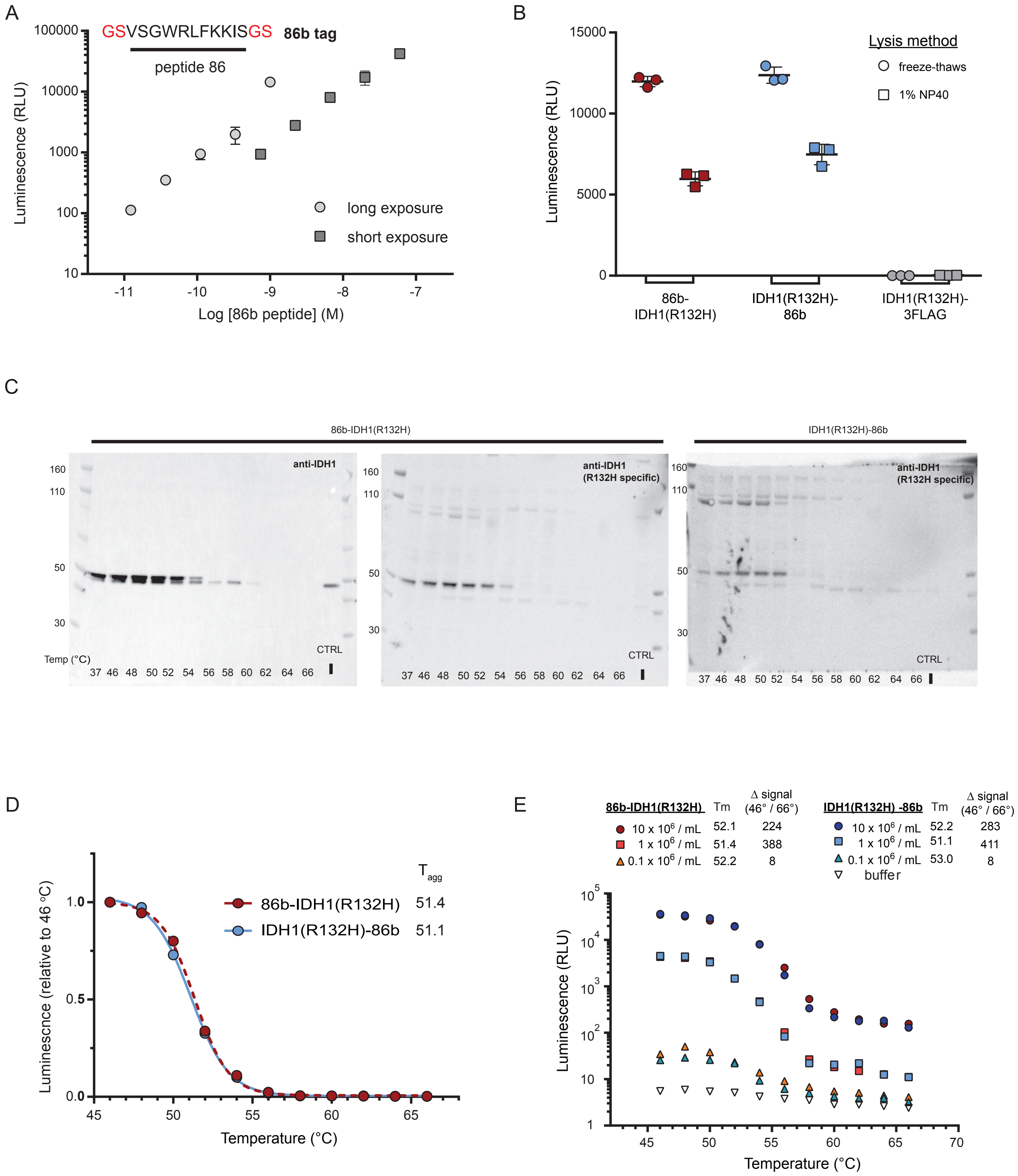

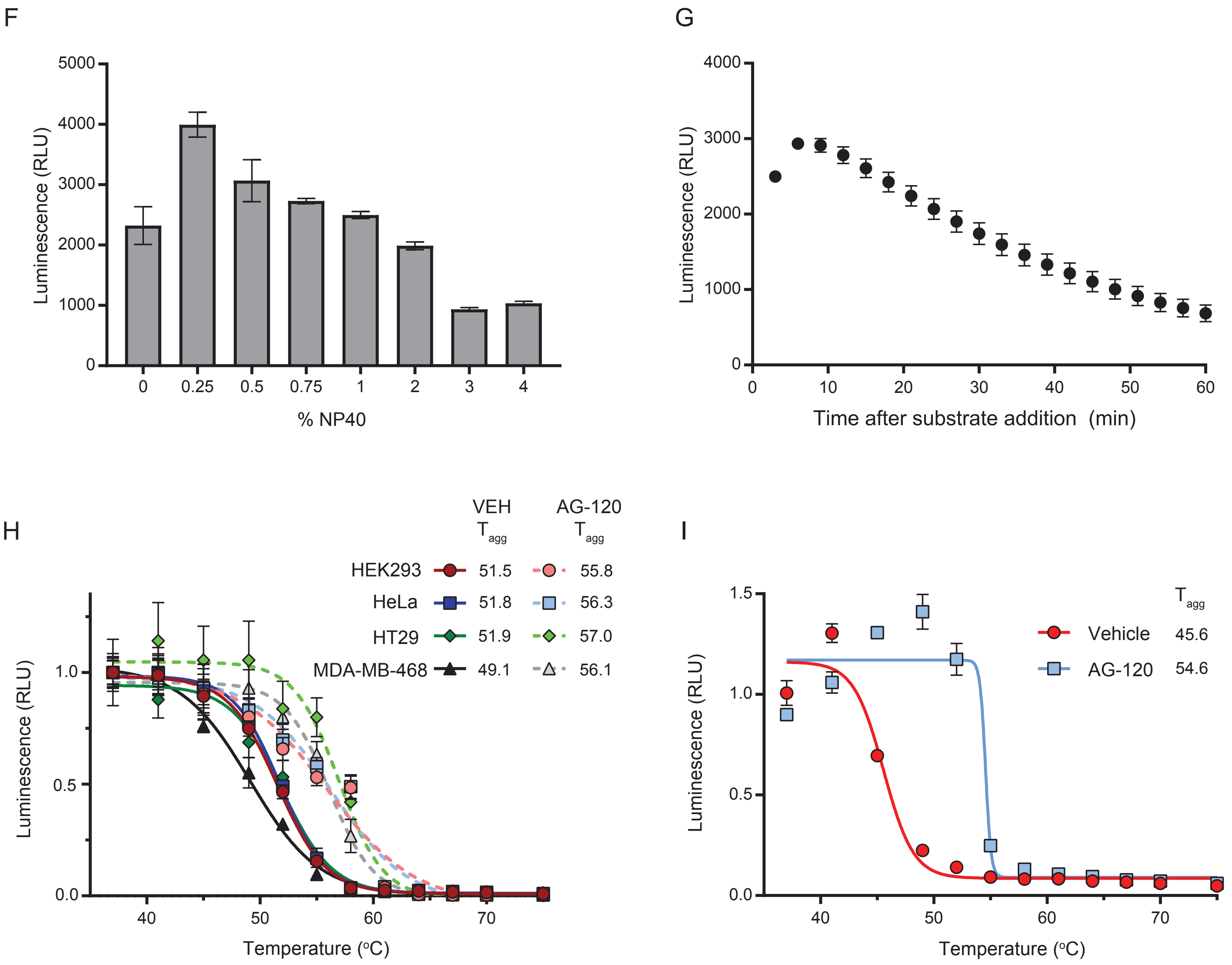

Supplementary Figure 1:

(A) Reconstitution of luciferase activity using purified fragments was tested in the presence of 100 nM 11S and 0.5X furimazine (mean ± SD, n=3). Two read settings were required to capture luminescence over the range of 86b peptide (mean ± SD, n=3) with a long exposure, 10 sec (R2 = 0.96) and a short exposure of 0.5 sec (R2=0.99). (B) HEK293T cells were transfected with 86b-tagged IDH1(R132H) or a FLAG-tagged control. After 24 h, cells were suspended in CETSA buffer at $2.5x10^6$ cells/mL and lysed by freeze-thaw cycles or 1% NP40. Reconstituted luciferase activity was assessed in the presence of 200 nM 11S fragment and 0.5X furimazine (n=3 per condition). (C) Uncropped western blots presented in Figure 1c. (D) Thermal stability of transfected 86b-tagged IDH1(R132H) examined by NanoLuc complementation assay for the same samples shown in Western blots in Fig. 1c,d. (E) IDH1(R132H) thermal profiles at different cell densities. Ratio of luminescence from lowest to highest temperature is shown in the inset. Note, the $1x10^6$/mL data is also presented in panel D. (F) Effect of NP40 on 86b/11S complementation. 100 nM 11S was mixed with 5 nM 86b peptide in CETSA buffer and NP40 concentration was adjusted between 0 and 4%. Luminescence was measured 5 min after adding 0.5X furimazine (mean ± SD, n= 3). (G) Kinetics of light emission for 5 nM 86b and 100 nM 11S in the presence of 1% NP40. Luminescence was measured over 60 minutes after adding 0.5X furimazine (mean ± SD, n= 3). (H) Treatment with 1 μM AG-120, an inhibitor of mutant IDH1, stabilizes C-tagged IDH1(R132H) in four cell lines. Cells were treated for 1 h and heated for 3.5 min (mean ± SD, n=4). (I) AG-120 stabilizes IDH1(R132H) when compound is added directly to lysates. HEK293T were transfected with IDH1(R132H)-86b and lysed in CETSA buffer containing 0.4% NP-40. AG-120 (1 μM) was added to lysate and samples were heated for 3.5 min (mean ± SD, n= 4).

Supplementary Figure 2

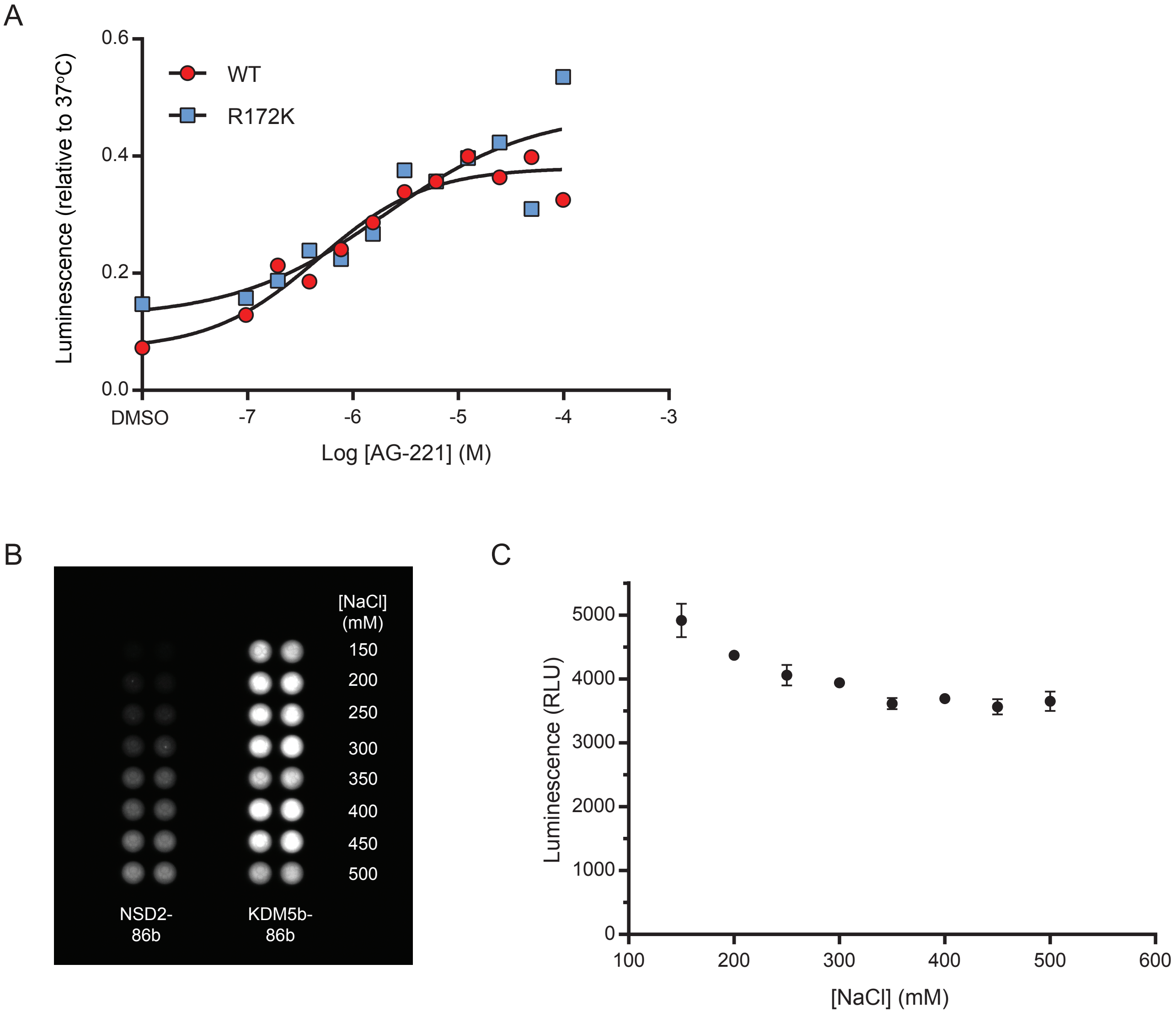

Supplementary Figure 2:

(A) AG-221 stabilizes both WT and IDH2(R172K) proteins (55 °C for 3.5 min). (B) High salt extraction increases recovery of some nuclear targets. NSD2-86b and KDM5b-86b were expressed in HEK293T cells and lysed in buffer containing NaCl ranging from 150 mM to 500 mM. (c) Effect of salt concentration on 86b/11S complementation. 100 nM 11S was mixed with 5 nM 86b peptide in CETSA buffer (DPBS with $CaCl_2$ and $MgCl_2$ + 1 g/L glucose + 1% NP40 + 0.5% DMSO + 1x protease inhibitors) and NaCl concentration was adjusted between 150 mM and 500 mM. Luminescence was measured 15 min after adding 0.5X furimazine (mean ± SD, n= 3).

Supplementary Figure 3

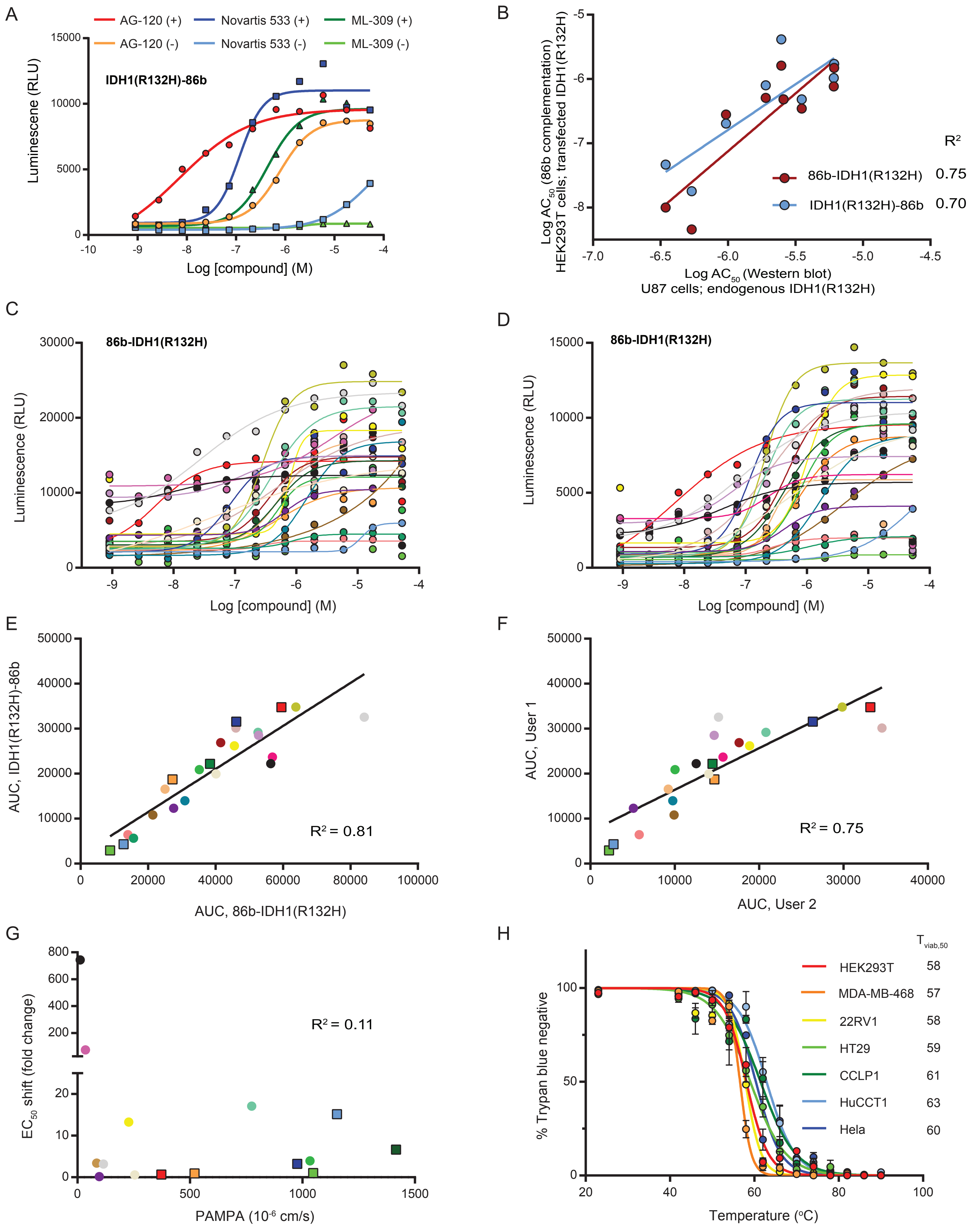

Supplementary Figure 3:

Analysis of SplitLuc CETSA data performed in a 384-well format (A) Thermal stabilization profile of C-terminal tagged IDH1(R132H) treated with three pairs of inhibitors (+, eutomer; -, distomer) and heated at 56 ^{o}C for 3.5 min. (B) Stabilization of IDH1(R132H)-86b with selected compounds correlates with traditional CETSA (Urban et al., 2017). (C) Individual thermal stabilization curves for N-tagged and (D) C-tagged IDH1(R132H) after 1 h treatment with 23 inhibitors ranging from 900 pM to 53 µM. Samples were heated to 56 ^{o}C for 3.5 min. Calculated AC_{50} values from the dose-response curves are presented in Fig. 3b. (E) Thermal stabilization of IDH1(R132H)-86b assessed by area under the curve, to assign a single value that incorporates both potency and magnitude of response. Squares indicate the compounds highlighted in Fig. 3a. (F) Good reproducibility was observed when experimental procedures were performed by different users. (G) PAMPA measurement of compound permeability (10^{-6} cm/sec) does not correlate with observed shift in potency when a wash step is included in the CETSA protocol. (H) Trypan blue exclusion assay in seven cell lines. After trypsinization and resuspension, cells were heated to various temperatures and percent trypan positive cells were counted using a hemocytometer (mean ± SD, n=2).

Supplementary Figure 4

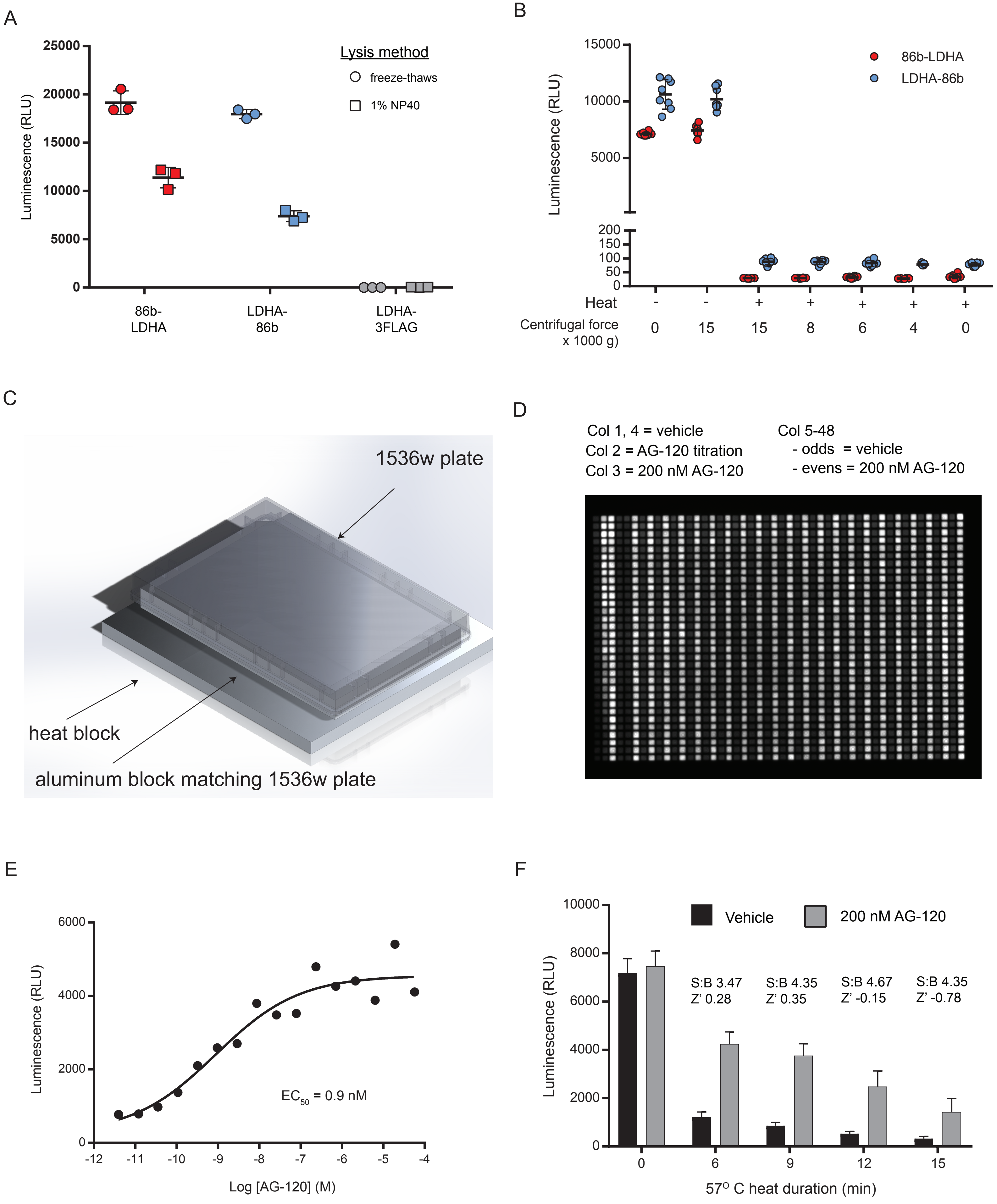

Supplementary Figure 4:

Optimization of the SplitLuc CETSA assay in a 1536-well format (A) HEK293T cells were transfected with 86b-tagged LDHA or a FLAG-tagged control. After 24 h, cells were suspended in CETSA buffer at $2.5x10^6$ cells/mL, lysed by freeze-thaw cycles or 1% NP40 and reconstitution of luciferase activity was assessed in the presence of 200 nM 11S fragment and 0.5X furimazine. (B) Centrifugation is not required for the removal of complementation incompetent LDHA-86b. After heating to 70 ^oC for 3.5 mins, samples were centrifuged for 20 min at various speeds and reconstituted NanoLuc was measured. (C) Schematic representation of custom-made aluminum block utilized to heat samples. (D) Stabilization of IDH1(R132H)-86b with 200 nM AG-120 in 1,536-well format. Transfected cells were dispensed into a 1,536-well plate and compound was added by pin tool transfer. After 1 h, the plate was heated at 57 ^oC for 9 min using an aluminum block. Reconstituted NanoLuc activity was detected using a ViewLux plate reader. (E) Dose-dependent stabilization of IDH1(R132H)-86b by AG-120 (57 ^oC for 9 min). (F) Plate statistics for vehicle DMSO vs. 200 nM AG-120 treated samples heated to 57 ^oC for different durations. Signal-to-background (S:B) indicates the fold change between groups treated with DMSO and compound. The Z-factor (Z') indicates the relative difference between positive and negative controls and their variances.

Supplementary Figure 5

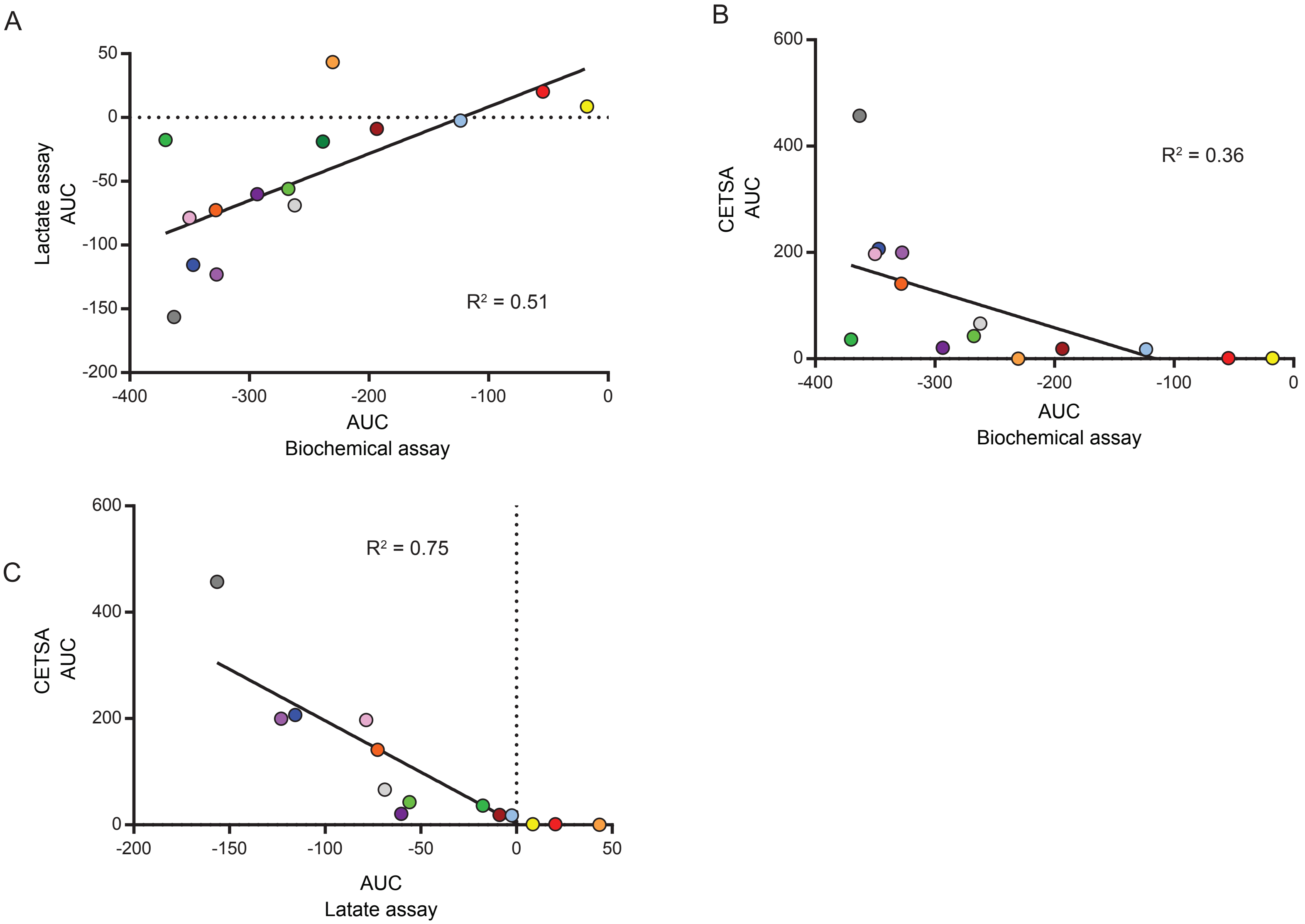

Supplementary Figure 5:
Correlation of area under the curve (response profile) for (A) biochemical vs. lactate, (B) biochemical vs. SplitLuc CETSA, and (C) SplitLuc CETSA vs. lactate production assays. Symbol colors match those presented in Fig. 5 and Supplementary Table 2. Dotted lines demark boundary between apparent inhibition and activation in the cellular lactate assay.

Supplementary Figure 6

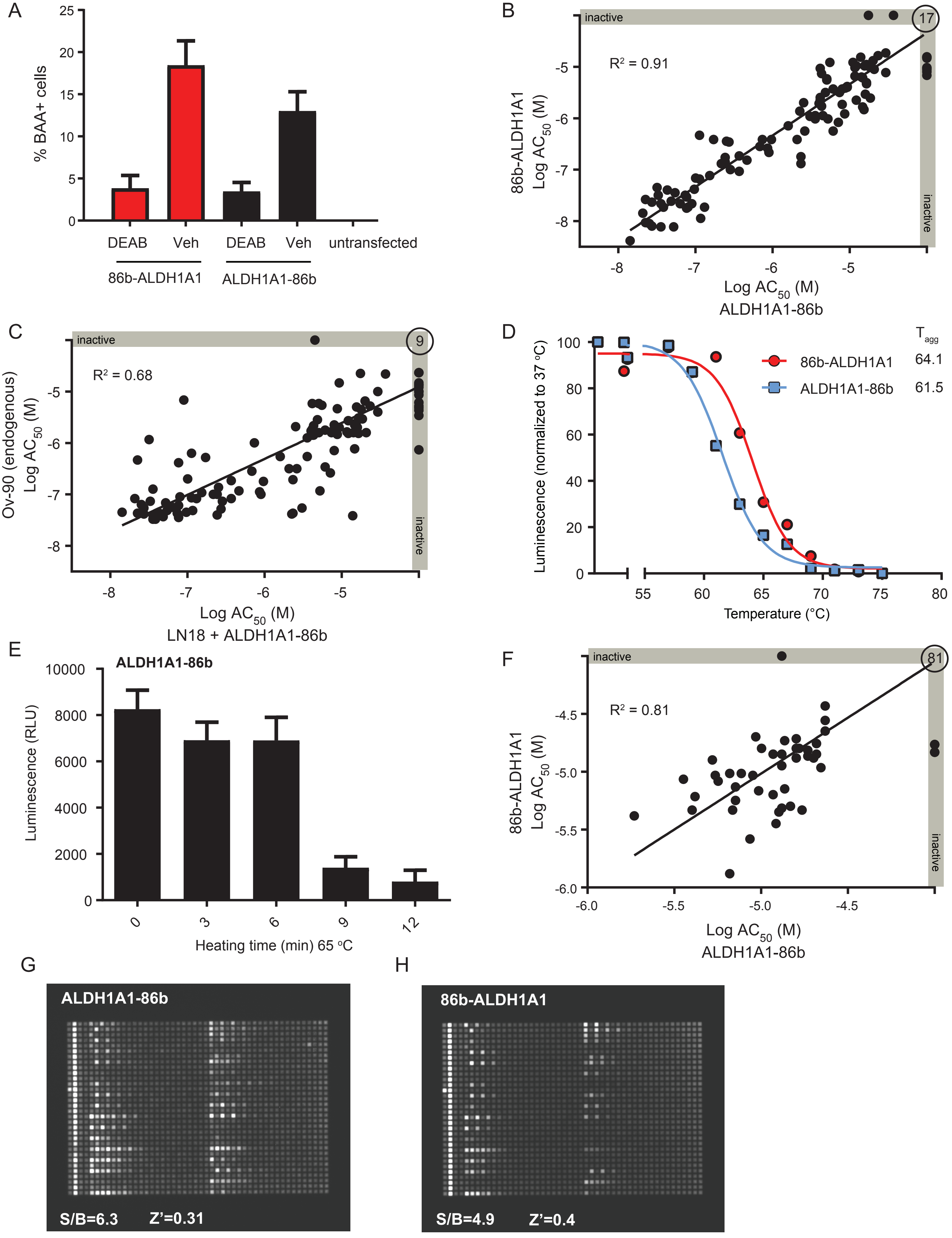

I

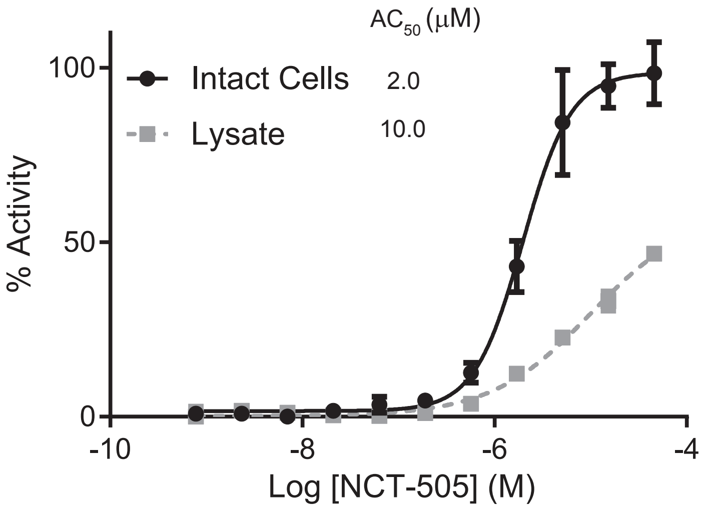

J

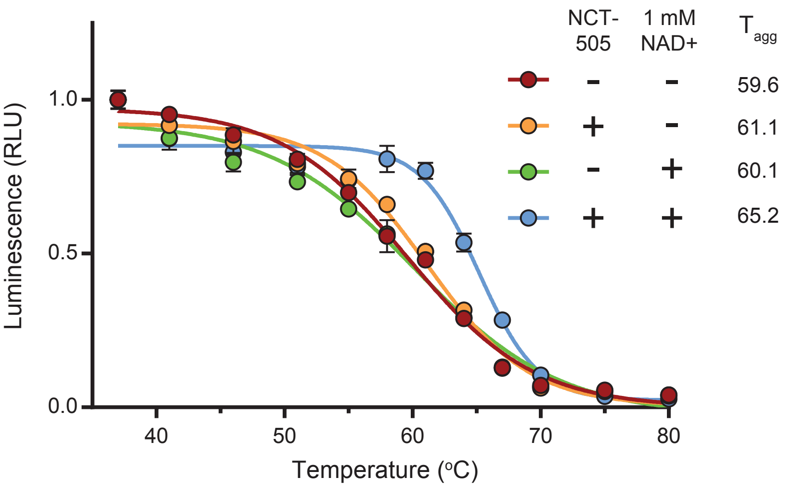

Supplementary Figure 6:

ALDH1A1 inhibitors (127 analogs) examined via SplitLuc-CETSA. (A) ALDH1A1 retains enzymatic activity when tagged with 86b at the N- and C-terminus. LN18 cells were transfected, dispensed into a 96-well plate and incubated for 24 h. ALDH1A1 activity was assessed using the Aldefluor high-content imaging assay in the presence of inhibitor (DEAB) or vehicle (DMSO). Data are represented as percent BAA positive cells, with BAA being the oxidized fluorescent product of ALDH1A1 enzymatic activity in the Aldefluor assay (mean ± SD, n= 8). (B) Inhibition of N- and C-tagged ALDH1A1 are highly correlated in transfected LN18 cells. The correlation plot indicates compound's LogAC$_{50}$ in the Aldefluor assay in 86b-ALDH1A1 (y-axis) and ALDH1A1-86b (x-axis) transfected LN18 cells. (C) Inhibition of endogenous ALDH1A1 activity (OV-90 cells) is correlated with inhibition of 86b-ALDH1A1 as measured by Aldefluor assay. The correlation plot indicates compound's LogAC$_{50}$ in the Aldefluor assay in Ov-90 (y-axis) and ALDH1A1-86b transfected LN18 cells (x-axis). (D) Thermal melt profiles for 86b N- and C-tagged ALDH1A1. (E) Luminescence from ALDH1A1-86b in 1536-well plates with different heat durations at 65 ^{o}C (mean ± SD, n= 320). (F) Thermal stabilization of N- and C-tagged ALDH1A1 in LN18 cells. Potency values (AC$_{50}$) were calculated for cells treated with 11 concentrations of each compound. 81 compounds were inactive in both assays. (G, H) Image of 1,536-well plate at the completion of the CETSA assay for (G) N-tagged or (H) C-tagged ALDH1A1. Controls are in columns 1-4. 11-point compound titrations move left to right in every other column (*i.e.* top concentration for compound A starts in column 5, compound B in column 6, compound C in column 26, compound D in column 27). (I) NCT-505 stabilization of ALDH1A1-86b was examined in intact LN18 cells versus lysates. The enyzme's cofactor NAD+ was added at 1 mM to lysates. Samples were heated in 1536-well plates for 9 min at 65 ^{o}C (mean ± SD, n=4). (J) ALDH1A1 inhibitor NCT-505 stabilizes ALDH1A1 in lysates only when 1 mM NAD+ is supplemented. LN18 cells were transfected with ALDH1A1-86b and lysed in CETSA buffer containing 0.4% NP-40 ± 1 mM NAD+. NCT-505 (30 μM) was added to lysate and samples were heated for 3.5 min (mean ± SD, n= 2).

Supplementary Figure 7

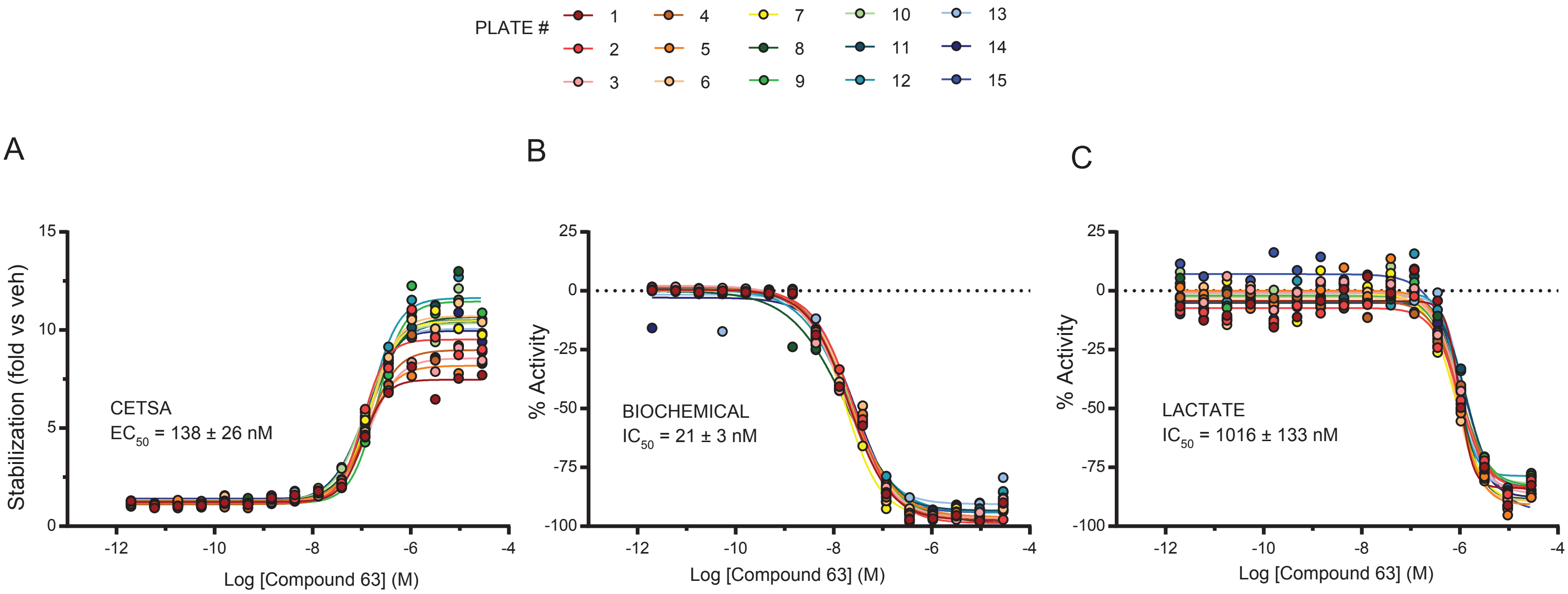

Supplementary Figure 7:

Performance of the control LDHA inhibitor Compound 63 across 15 plates of the MIPE screen. Dose-response curves for the control compound for (A) SplitLuc CETSA, (B) biochemical, and (C) lactate production screens. Potency +/- SD values are indicated for the control inhibitor, calculated across all plates from the respective screens.

Supplementary Figure 8

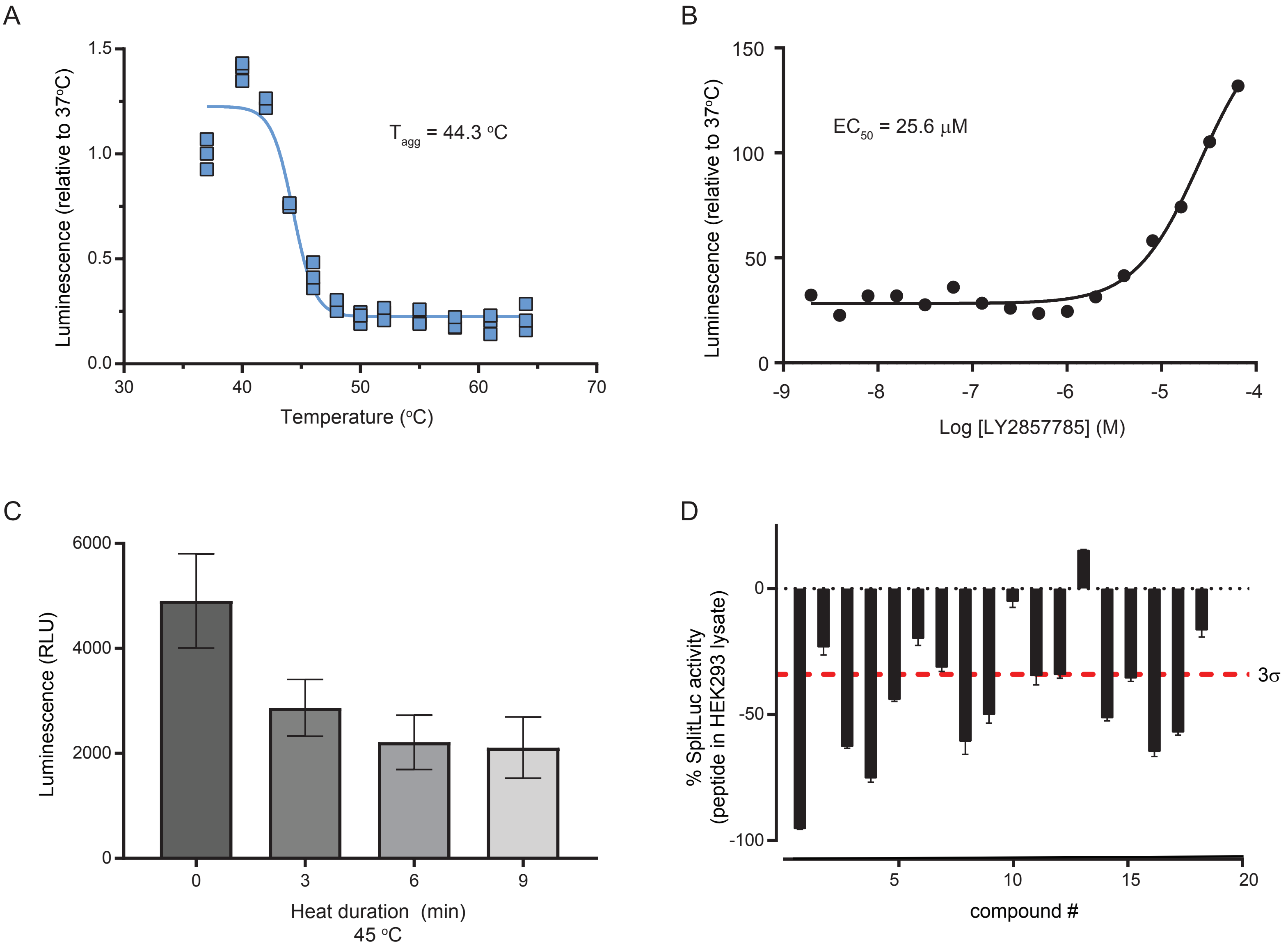

Supplementary Figure 8:

CDK9 assay optimization. (A) Thermal stability of transfected CDK9-86b examined by SplitLuc CETSA. Cells were assayed after 48 h transfection. (B) Stabilization of CDK9-86b by the CDK9 inhibitor LY2857785. (C) Luminescence from CDK9-86b in 1536-well plates with different heat durations at 45 °C (mean ± SD, n= 896 wells per group). (D) 18 compounds found as destabilizers in the CDK9-86b qHTS (kinase inhibitor collection) were tested at 46 μM in a counterscreen using parental (untransfected) HEK293T cell lysate supplemented with 86b peptide. Dotted line indicates 3xSD from the mean of vehicle controls.

Supplementary Table 1: 86b tagged targets presented in this study.

Target	Full Name	Uniprot ID	Molecular function	Size (a.a., untagged)	Subcellular localization	Variant(s)	Cloning approach	Cloning oligonucleotides	Notes
ALDH1A1	Retinal dehydrogenase 1	P00352	oxidoreductase	501	cytoplasm	WT	Gene synthesis	n/a	
CDK9	Cyclin-dependent kinase 9	P50750	kinase	372	nucleus	WT	In-Fusion	for: 5'-ACCCAAGCTGGCTAGCCACCATGGCGAAGCAGTACGACTC-3'<br>rev: 5'-AGCCACTCACGGATCCGAAGACGCGCTCAAACTCCG-3'	
CFTR	Cystic fibrosis transmembrane conductance regulator	P13569	chloride channel	1480 (wt)<br>1479 (ΔF508)	endoplasmic reticulum and plasma membrane	WT and ΔF508 mutant<br>both contain V370M substitution	In-Fusion	for: 5'-ACCCAAGCTGGCTAGCCACCATGCAGAGGTCGCCTCTGG-3'<br>rev: 5'-AGCCACTCACGGATCCAAGCCTTGTATCTTGCACCTCTTC-3'	
DHFR	Dihydrofolate reductase	P00374	oxidoreductase	187	mitochondria, cytoplasm	WT	Gene synthesis	n/a	
HDAC1	Histone deacetylase 1	Q13547	histone deacetylase	482	nucleus	WT	In-Fusion	for: 5'-ACCCAAGCTGGCTAGCCACCATGGCGCAGACGCAGG-3'<br>rev: 5'-AGCCACTCACGGATCCGGCCAACTTGACCTCCTCCT-3'	
GBA	Beta-glucocerebrosidase (GC)	P04062	glucosylceramidase	536	lysosome	N370S mutant	Gene synthesis	n/a	86b tag placement after signal peptide
IDH1	Isocitrate dehydrogenase [NADP] cytoplasmic	O75874	oxidoreductase	414	cytoplasm, peroxisome	R132H mutant	Gene synthesis	n/a	
IDH2	Isocitrate dehydrogenase [NADP], mitochondrial	P48735	oxidoreductase	452	mitochondria	WT and R172K mutant	Gateway	n/a	Addgene #81800 (wt)<br>Addgene #81432 (R172K)
KDM5B	Lysine-specific demethylase 5B	Q9UGL1	Fe2+ dependent demethylase	1544	nucleus	WT	In-Fusion	for: 5'-ACCCAAGCTGGCTAGCCACCATGGAGGCGGCCACCAC-3'<br>rev: 5'-AGCCACTCACGGATCCCTTTCGGCTTGGTGCGTCCTT-3'	
LDHA	L-lactate dehydrogenase A chain	P00338	oxidoreductase	332	cytoplasm	WT	Gene synthesis	n/a	
NSD2	Histone-lysine N-methyltransferase NSD2	O96028	histone methyltransferase	1365	nucleus	WT	In-Fusion	for: 5'-ACCCAAGCTGGCTAGCCACCATGGAATTTAGCATCAAGCAGAGTCCC-3'<br>rev: 5'-AGCCACTCACGGATCCTTTGCCCTCTGTGACTCTCC-3'	

Supplementary Table 2: Summary of LDHA inhibitors. Symbol colors match data presented in Figures 5 and S5.

Symbol	NCGC ID	Compound # Rai et al. J Med Chem	Structure	Biochemical Assay		Lactate production (HEK293T)		CETSA LDHA-86b (HEK293T)	
				IC50	Efficacy	IC50	Efficacy	EC50	Efficacy
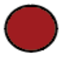	NCGC00263436-01	1	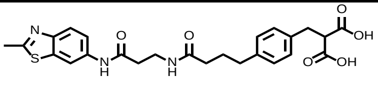 CC1=NC2=CC=C(NC(=O)CCNC(=O)CCCC3=CC=C(CC(C(O)=O)C(O)=O)C=C3)C=C2S1	1.19	-85.9	inactive	n/a	16.53	49.3
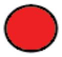	NCGC00274266-04	5	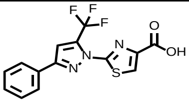 OC(=O)C1=CSC(=N1)[N]2N=C(C=C2C(F)(F)F)C3=CC=CC=C3	33.54	-37.3	inactive	22.302	inactive	n/a
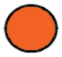	NCGC00355875-01	2	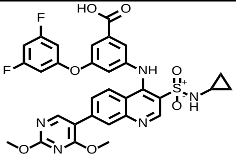 COC1=NC(=C(C=N1)C2=CC=C3C(=C(C=NC3=C2)[S+]([O-])(=O)NC4CC4)NC5=CC(=CC(=C5)C(O)=O)OC6=CC(=CC(=C6)F)F)C	0.05	-97.0	19.18224	-74.447	1.65	90.2
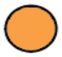	NCGC00355887-03	33	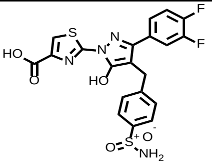 N[S+]([O-])(=O)C1=CC=C(CC2=C(O)[N](N=C2C3=CC=C(F)C(=C3)F)C4=NC(=CS4)C(O)=O)C=C1	0.60	-95.1	inactive	n/a	inactive	n/a
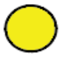	NCGC00356269-01	34	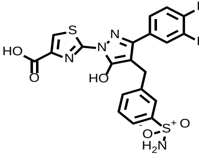 N[S+]([O-])(=O)C1=CC=CC(=C1)CC2=C(O)[N](N=C2C3=CC=C(F)C(=C3)F)C4=NC(=CS4)C(O)=O	inactive	n/a	inactive	n/a	inactive	n/a
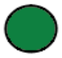	NCGC00356387-02	37	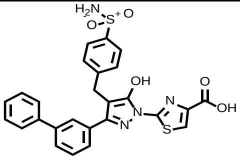 N[S+]([O-])(=O)C1=CC=C(CC2=C(O)[N](N=C2C3=CC=CC(=C3)C4=CC=CC=C4)C5=NC(=CS5)C(O)=O)C=C1	0.27	-96.7	inactive	n/a	inactive	n/a
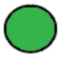	NCGC00356705-02	44	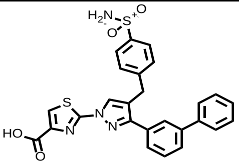 N[S+]([O-])(=O)C1=CC=C(CC2=C[N](N=C2C3=CC(=CC=C3)C4=CC=CC=C4)C5=NC(=CS5)C(O)=O)C=C1	0.04	-103.2	6.806115	-58.374	20.82	105.8
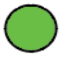	NCGC00356756-05	57	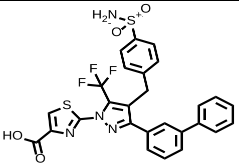 N[S+]([O-])(=O)C1=CC=C(CC2=C([N](N=C2C3=CC(=CC=C3)C4=CC=CC=C4)C5=NC(=CS5)C(O)=O)C(F)(F)F)C=C1	0.67	-147.5	10.78697	-62.434	13.13	83.0
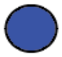	NCGC00357537-02	61	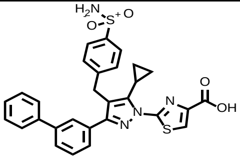 N[S+]([O-])(=O)C1=CC=C(CC2=C(C3CC3)[N](N=C2C4=CC=CC(=C4)C5=CC=CC=C5)C6=NC(=CS6)C(O)=O)C=C1	0.02	-94.2	1.078697	-56.889	2.62	151.8

	NCGC00371840-01	69	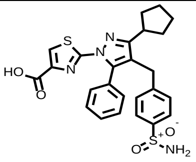 N[S+]([O-])(=O)C1=CC=C(CC2=C([N](N=C2C3CCCC3)C4=NC(=CS4)C(O)=O)C5=CC=CC=C5)C=C1	6.69	-86.9	inactive	n/a	20.82	22.3
	NCGC00371904-01	70	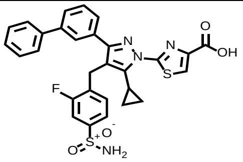 N[S+]([O-])(=O)C1=CC=C(CC2=C(C3CC3)[N](N=C2C4=CC=CC(=C4)C5=CC=CC=C5)C6=NC(=CS6)C(O)=O)C(=C1)F	0.04	-91.3	1.709619	-58.079	2.08	135.8
	NCGC00371908-01	60	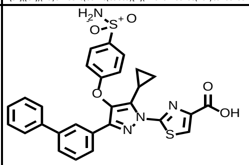 N[S+]([O-])(=O)C1=CC=C(OC2=C(C3CC3)[N](N=C2C4=CC=CC(=C4)C5=CC=CC=C5)C6=NC(=CS6)C(O)=O)C=C1	0.08	-91.9	6.065956	-61.035	18.55	55.0
	NCGC00371956-01	62	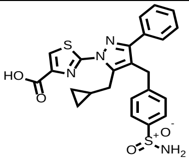 N[S+]([O-])(=O)C1=CC=C(CC2=C(CC3CC3)[N](N=C2C4=CC=CC=C4)C5=NC(=CS5)C(O)=O)C=C1	0.03	-125.6	4.294368	-62.659	5.87	197.7
	NCGC00372530-03	63	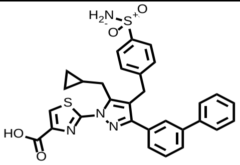 N[S+]([O-])(=O)C1=CC=C(CC2=C(CC3CC3)[N](N=C2C4=CC(=CC=C4)C5=CC=CC=C5)C6=NC(=CS6)C(O)=O)C=C1	0.03	-98.3	0.707946	-93.665	0.37	242.0
	NCGC00420796-02	3	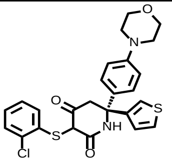 ClC1=CC=CC=C1SC2C(=O)C[C@](NC2=O)(C3=CSC=C3)C4=CC=C(C=C4)N5CCOCC5	0.30	-99.4	3.411138	-52.361	18.55	148.4

* compounds 69 and 70 not presented in Rai et al.

Supplementary Table 3: Summary of 89 actives identified in CDK9 Lanthascreen.

Sample ID	Sample Name	Structure	Supplier ID	Supplier	AC50 (nM) [a]	Curve Class [b]	Efficacy [c]	Max Resp	Log AC50 (M)	Active in CETSA	Previously reported CDK9 activity
NCGC00378804-01	CDK-IN-2	COC1=C(C=C(F)C=C1)C2=CC(=NC=C2Cl)NC(=O)[C@@H]3CCCNC3	HY-13033	MedChem Express	null	5	0.0	-98.0	null	X	X
NCGC00242047-05	GW779439X	CN1CCN(CC1)C2=C(C=C(NC3=NC(=CC=N3)C4=C5C=CC=N[N]5N=C4)C=C2)C(F)(F)F	GLXC-03131	Glixx	null	5	0.0	-94.4	null		
NCGC00387028-01	PIK-75	CN(\N=C/C1=CN=C2C=CC(=C[N]12)Br)[S+]([O-])(=O)C3=C(C)C=CC(=C3)[N+]([O-])=O	SYN-1067	SynKinase	null	5	0.0	-93.4	null		
NCGC00389259-02	OTSSP167	CN(C)CC1CCC(CC1)NC2=C3NC(\C=C/C3=NC=C2C(C)=O)=C\4/C=C(Cl)\C(=O)\C(=C4)Cl	A12851	Adooq	1.1	-1.1	-82.5	-98.5	-9.0		
NCGC00481606-01	AZD-5597	CN[C@H]1CCN(C1)C(=O)C2=CC=C(NC3=NC=C(F)C(=N3)C4=CN=C(C)[N]4C(C)C)C=C2	B5926	APExBIO	1.9	-1.3	-51.7	-96.5	-8.7		X
NCGC00346500-05	PIK-75	CN(/N=C/C1=CN=C2C=CC(=C[N]12)Br)[S+]([O-])(=O)C3=C(C)C=CC(=C3)[N+]([O-])=O	CT-PIK75	ChemieTek	3.0	5	-24.5	-95.2	-8.5		
NCGC00346940-02	CGP60474	OCCCNC1=CC(=CC=N1)C2=NC(=NC=C2)NC3=CC=CC(=C3)Cl	HY-11009	MedChem Express	8.4	-1.2	-56.9	-98.0	-8.1		
NCGC00389231-01	P276-00	CN1CCC(C1CO)C2=C(O)C=C(O)C3=C2O\C(=C/C3=O)C4=CC=CC=C4Cl	B1430	APExBIO	8.4	-1.2	-60.7	-99.0	-8.1		X
NCGC00345809-02	Cdk/Crk Inhibitor	CC(C)C1=N[N](C\2=C1C(=O)N\C(=N2)CC3=CC=C(OCCO)C=C3)C4=C(Cl)C=CC=C4Cl	CD0252	Chemdea	10.6	-1.2	-57.1	-99.2	-8.0		X
NCGC00389551-02	Flavopiridol	CN1CCC(C(O)C1)C2=C(O)C=C(O)C3=C2O\C(=C/C3=O)C4=CC=CC=C4Cl	2090	Bio Vision	13.4	5	-38.0	-96.7	-7.9		X
NCGC00378999-05	SB1317	CN1C/C=C/CCOC2=CC=CC(=C2)C3=CC=NC(=N3)NC4=CC=CC(=C4)C1	HY-15166	MedChem Express	15.0	5	-6.2	-95.8	-7.8	X	X
NCGC00345852-02	BS194	CC(C)C1=C2N=C(N[C@@H](CO)[C@H](O)CO)C=C(NCC3=CC=CC=C3)[N]2N=C1	SYN-1151	SynKinase	16.8	-1.1	-108.2	-95.8	-7.8		X
NCGC00475726-01	BAY-1143572	COC1=CC(=CC=C1C2=NC(=NC=N2)NC3=CC(=CC=C3)C[S+](C)([O-])=N)F	A-1514	ActiveBioChem	21.2	-1.2	-55.3	-100.3	-7.7	X	X
NCGC00390459-03	Alsterpaullone	[O-][N+](=O)C1=CC2=C([NH]C3=C2CC(=O)NC4=CC=CC=C34)C=C1	A4847	SIGMA	26.0	-1.1	-82.6	-92.4	-7.6		X
NCGC00379060-01	NVP-LCQ195	C[S+]([O-])(=O)N1CCC(CC1)NC(=O)C2=C(NC(=O)C3=C(Cl)C=CC=C3Cl)C=N[NH]2	HY-15241	MedChem Express	29.9	-1.2	-72.2	-98.8	-7.5		X
NCGC00387038-01	GDC-046	ClC1=C(C(=O)NC2=CC(=NC=C2)NC(=O)C3CC3)C(=CC=C1)Cl	SYN-1198	SynKinase	33.6	-1.1	-83.7	-94.2	-7.5		
NCGC00092293-15	Ro 31-8220 (mesylate)	C[N]1C=C(C2=C1C=CC=C2)C/3=C(/C(=O)NC3=O)C4=C[N](CCCSC(N)=N)C5=C4C=CC=C5	HY-13866	MedChem Express	37.7	-1.3	-42.0	-60.6	-7.4		
NCGC00386340-02	LDC000067	COC1=CC=CC=C1C2=NC=NC(=C2)NC3=CC(=CC=C3)C[S+](N)([O-])=O	HY-15878	MedChem Express	47.4	-1.1	-96.3	-94.5	-7.3		X
NCGC00481577-01	TP-0903	CN(C)[S+]([O-])(=O)C1=CC=CC=C1\N=C/2N\C(=N/C=C2Cl)NC3=CC=C(CN4CCN(C)CC4)C=C3	B5940	APExBIO	68.7	-1.2	-74.0	-96.9	-7.2		
NCGC00351599-06	LY2835219 Mesylate	CCN1CCN(CC1)CC2=CN=C(NC3=NC=C(F)C(=N3)C4=CC(=C5N=C(C)[N](C(C)C)C5=C4)F)C=C2	GLXC-90003	Glixx	75.2	-1.3	-45.8	-96.3	-7.1		X
NCGC00346632-06	R547	COC1=CC=C(F)C(=C1C(=O)C2=C(N)N=C(NC3CCN(CC3)[S+](C)([O-])=O)N=C2)F	HY-10014	MedChem Express	75.2	5	-13.4	-94.3	-7.1		
NCGC00387501-01	C16	O=C\1NC2=CC=C3N=CSC3=C2C1=C/C4=C[NH]C=N4	5382	Tocris	84.4	-1.2	-77.3	-68.5	-7.1		
NCGC00390634-01	LY2857785	CC(C)C1=C2C=C(C=CC2=N[N]1C)C3=NC(=NC=C3)N[C@H]4CC[C@@H](CC4)NC5CCOCC5	HY-12293	MedChem Express	84.4	5	-28.1	-89.7	-7.1		X
NCGC00250410-12	GSK-650394	OC(=O)C1=C(C=C(C=C1)C2=C[NH]C3=NC=C(C=C23)C4=CC=CC=C4)C5CCCC5	SML0773	SIGMA	94.7	-1.2	-77.0	-89.0	-7.0		
NCGC00263200-02	RO495	CC1=NC(=NC(=C1)NC2=CC(=CC=N2)NC(=O)C3=C(Cl)C=CC=C3Cl)N	SYN-1128	SynKinase	94.7	-1.1	-93.8	-99.1	-7.0		
NCGC00263091-15	AT7519	ClC1=C(C(=O)NC2=C[NH]N=C2C(=O)NC3CCNCC3)C(=CC=C1)Cl	HY-50940A	MedChem Express	94.7	5	-14.9	-96.9	-7.0		X
NCGC00346617-09	AZD5438	CC(C)[N]1C(=NC=C1C2=NC(=NC=C2)NC3=CC=C(C=C3)[S+](C)([O-])=O)C	GLXC-04428	Glixx	106.2	-1.2	-75.7	-98.3	-7.0		X
NCGC00263151-08	JNJ7706621	NC1=NC(=N[N]1C(=O)C2=C(F)C=CC=C2F)NC3=CC=C(C=C3)[S+](N)([O-])=O	GLXC-90017	Glixx	106.2	-1.1	-93.4	-97.5	-7.0		
NCGC00346827-02	UCN-01	CN[C@@H]1C[C@H]2O[C@@](C)([C@@H]1OC)[N]3C4=C(C=CC=C4)C5=C6[C@H](O)NC(=O)C6=C7C8=C(C=CC=C8)[N]2C7=C35	U6508	SIGMA	108.8	-1.1	-83.5	-93.0	-7.0		
NCGC00346658-09	CP-868596 (Crenolanib)	CC1(COC1)COC2=CC3=C(C=C2)[N](C=N3)C4=NC5=C(C=CC=C5C=C4)N6CCC(N)CC6	GLXC-90124	Glixx	119.2	-1.1	-91.6	-90.2	-6.9		
NCGC00371153-09	RKI-1447	OC1=CC(=CC=C1)CNC(=O)NC2=NC(=CS2)C3=CC=NC=C3	GLXC-05200	Glixx	133.7	-1.2	-64.8	-59.5	-6.9		
NCGC00346677-07	CHIR98014	NC1=NC(=CC=C1[N+]([O-])=O)NCCNC2=NC=C([N]3C=CN=C3)C(=N2)C4=CC=C(Cl)C=C4Cl	GLXC-05956	Glixx	133.7	-1.1	-87.0	-80.6	-6.9		
NCGC00263168-06	PHA-793887	CC(C)CC(=O)NC1=N[NH]C2=C1CN(C(=O)C3CCN(C)CC3)C2(C)C	HY-11001	MedChem Express	133.7	-1.1	-87.0	-99.5	-6.9		X
NCGC00096117-01	5-Iodotubercidin	NC1=NC=NC2=C1C(=C[N]2[C@@H]3O[C@H](CO)[C@H](O)C3O)I	KoLibri-I-004	Biolog	150.0	-1.1	-92.5	-99.1	-6.8		
NCGC00242495-18	PF-562271	CN(C1=NC=CC=C1CNC2=NC(=NC=C2C(F)(F)F)NC3=CC=C4NC(=O)CC4=C3)[S+](C)([O-])=O	HY-10459	MedChem Express	150.0	-1.1	-92.3	-101.3	-6.8		
NCGC00346945-06	BAY 61-3606 (dihydrochloride)	COC1=CC=C(C=C1OC)C2=CC3=NC=C[N]3C(=N2)NC4=NC=CC=C4C(N)=O	HY-14985	MedChem Express	150.0	-1.1	-91.8	-92.1	-6.8		X
NCGC00263147-05	A-674563	CC1=N[NH]C2=CC=C(C=C12)C3=CC(=CN=C3)OC[C@@H](N)CC4=CC=CC=C4	HY-13254	MedChem Express	150.0	-1.1	-89.8	-98.7	-6.8		
NCGC00346718-05	CP-673451	COCCOC1=CC=C2[N](C=NC2=C1)C3=NC4=C(C=CC=C4C=C3)N5CCC(N)CC5	HY-12050	MedChem Express	150.0	-1.1	-83.3	-91.2	-6.8		
NCGC00347278-02	A-443654	CC1=N[NH]C2=C1C=C(C=C2)C3=CN=CC(=C3)OC[C@@H](N)CC4=C[NH]C5=C4C=CC=C5	HY-10425	MedChem Express	168.3	-1.1	-88.0	-96.6	-6.8		
NCGC00241412-08	kb-NB142-70	OC1=CC=C2SC3=C(SCCNC3=O)C2=C1	SML0525	SIGMA	168.3	-1.1	-82.7	-91.1	-6.8		
NCGC00384250-01	NCGC00384250-01	FC1(F)CCN(C1)C2=CC(=CC(=N2)NC3=CC(=CC=N3)C#N)C4CCN(CC4)C5COC5	GNE-3511	ChemoGenics	188.9	-1.2	-76.1	-87.1	-6.7		
NCGC00389697-03	AT9283	O=C(NC1CC1)NC/2=C/NNC2=C/3\N=C4\C=C/C(=C\C4=N3)CN5CCOCC5	2432	Bio Vision	188.9	-1.1	-93.9	-97.0	-6.7		
NCGC00244257-08	CYT-387	O=C(NCC#N)C1=CC=C(C=C1)C2=NC(=NC=C2)NC3=CC=C(C=C3)N4CCOCC4	CT-CYT387	ChemieTek	188.9	-1.1	-85.8	-96.7	-6.7		
NCGC00346954-01	TCS-2312	OC1=CC=C(C(=C1)O)C2=CC=C(C=C2)C3=N[NH]C(=C3)NC4=CC=CC(=C4)CNC5CC5	3038	Tocris	188.9	-1.1	-85.0	-94.1	-6.7		
NCGC00386425-06	Defactinib (VS-6063, PF-04554878)	CNC(=O)C1=CC=C(NC2=NC(=C(C=N2)C(F)(F)F)NCC3=C(N=CC=N3)N(C)[S+](C)([O-])=O)C=C1	GLXC-90041	Glixx	188.9	-1.1	-79.8	-50.3	-6.7		
NCGC00379187-01	PPY A	COC1=C(C=CC=C1)C2=C[NH]C3=NC=C(C=C23)C4=CC(=CN=C4)C(=O)N(C)C	4730	Tocris	237.8	-1.1	-90.1	-95.0	-6.6		
NCGC00390623-02	Pacritinib	C1CCN(C1)CCOC2=C3COC/C=C/COCC4=CC=CC(=C4)C/5=N/C(=N\C(=C3)C=C2)N/C=C5	HY-16379	MedChem Express	299.3	-1.2	-64.6	-95.3	-6.5		
NCGC00389549-01	LY2874455	CC(OC1=CC2=C([NH]N=C2\C=C\C3=C[N](CCO)N=C3)C=C1)C4=C(Cl)C=NC=C4Cl	A3576	APExBIO	299.3	-1.1	-87.6	-91.8	-6.5		
NCGC00379125-02	A 1070722	COC1=CC=C2C(=CC=NC2=C1)NC(=O)NC3=NC(=CC=C3)C(F)(F)F	1909	Axon Medchem	335.9	-1.4	-58.3	-64.1	-6.5		
NCGC00387772-02	AS601245	N#C\C(=C\1NC2=CC=CC=C2S1)C3=NC(=NC=C3)NCCC4=CC=CN=C4	2162	Bio Vision	335.9	-1.2	-79.8	-85.8	-6.5		
NCGC00387161-01	TC-DAPK 6	O=C1OC(=N/C1=C\C2=CC=CN=C2)\C=C\C3=CC=CC=C3	4301	Tocris	335.9	-1.2	-71.2	-68.2	-6.5		
NCGC00345739-02	KH-CB19	CCOC(=O)C1=C(\C(=C/N)C#N)C2=CC=C(Cl)C(=C2[N]1C)Cl	4262	Tocris	335.9	-1.1	-88.2	-90.2	-6.5		
NCGC00015582-02	Kenpaullone	BrC1=CC2=C([NH]C3=C2CC(=O)NC4=C3C=CC=C4)C=C1	Tocris-1398	Tocris	335.9	-1.1	-80.5	-37.7	-6.5		
NCGC00386926-01	null	[O-][N+](=O)C1=CC=CC2=C1N=C(CNC3=NC(=NC4=C3N=C[N]4C5=CSC=C5)N6CCOCC6)[NH]2	MLS003128609	Evotec	376.9	-1.2	-69.1	-72.6	-6.4		
NCGC00378978-05	XL-228	CC(C)C1=NOC(=C1)CNC2=NC(=CC(=N2)NC3=N[NH]C(=C3)C4CC4)N5CCN(C)CC5	GLXC-06661	Glixx	376.9	-1.1	-97.1	-96.7	-6.4		
NCGC00263129-21	Palbociclib (PD-0332991) HCl	CC(=O)C\1=C(/C)C2=CN=C(NC3=CC=C(C=N3)N4CCNCC4)N=C2N(C5CCCC5)C1=O	GLXC-90168	Glixx	376.9	-1.1	-92.3	-91.0	-6.4		
NCGC00379020-03	JNK-IN-7	CN(C)C/C=C/C(=O)NC1=CC=CC(=C1)C(=O)NC2=CC=C(NC3=NC=CC(=N3)C4=CN=CC=C4)C=C2	A3519	APExBIO	376.9	-1.1	-86.0	-91.6	-6.4		
NCGC00263020-01	NCGC00263020-01	CC(C)[N]1C=C(C2=CC=NC(=N2)NC3=C(C)C=CC(=C3)C(N)=O)C4=C1C=NC=C4	JKJ22-049	NCGCChem	398.1	-1.1	-90.3	-99.8	-6.4		
NCGC00263204-10	CYC-116	CC1=C(SC(=N1)N)C2=NC(=NC=C2)NC3=CC=C(C=C3)N4CCOCC4	GLXC-90172	Glixx	422.8	-1.1	-92.0	-90.5	-6.4		
NCGC00388443-01	CAY10574	NC1=C(\N=N/C2=CC=C(O)C=C2)C(=N[NH]1)N	10011247	Cayman	422.8	-1.1	-84.6	-95.5	-6.4		X

NCGC00485610-01	GW8510	[O-][S+](=O)(NC1=NC=CC=C1)C2=CC=C(N/C=C/3C(=O)NC4=C3C5=C(C=C4)N=CS5)C=C2	G7791	SIGMA	433.2	-1.1	-99.4	-92.5	-6.4		
NCGC00250381-03	AZ 23	CC(C)OC1=CC(=N[NH]1)NC2=C(Cl)C=NC(=N2)N[C@@H](C)C3=CC=C(F)C=N3	1610	Axon Medchem	474.4	-1.2	-76.1	-97.4	-6.3		
NCGC00250386-12	BX-795 hydrochloride	IC1=C(NCCCNC(=O)C2=CC=CS2)N=C(NC3=CC(=CC=C3)NC(=O)N4CCCC4)N=C1	SML0694	SIGMA	474.4	-1.1	-99.6	5.8	-6.3		
NCGC00016027-04	Tyrphostin AG 537	OC1=C(O)C=C(C=C1)\C=C(/C#N)C(=O)NCCCNC(=O)\C(=C\C2=CC(=C(O)C=C2)O)C#N	Lopac-T-4693	SigmaAldrich	474.4	-1.1	-98.0	-83.8	-6.3		
NCGC00485937-01	UNC2881	CCCCNC1=NC(=C(C=N1)C(=O)NCC2=CC=C(C=C2)[N]3C=CN=C3)N[C@H]4CC[C@@H](O)CC4	HY-15798	MedChem Express	474.4	-1.1	-93.2	-99.2	-6.3		
NCGC00402345-02	VX-509 (Decernotinib)	CC[C@@](C)(NC1=CC=NC(=N1)C2=C[NH]C3=C2C=CC=N3)C(=O)NCC(F)(F)F	GLXC-90220	Glixx	474.4	-1.1	-91.7	-97.1	-6.3		
NCGC00093787-06	DMAT	CN(C)C1=NC2=C(Br)C(=C(Br)C(=C2[NH]1)Br)Br	HY-15535	MedChem Express	474.4	-1.1	-84.2	-94.6	-6.3		
NCGC00379012-01	CVT-313	COC1=CC=C(CNC2=C3N=C[N](C(C)C)C3=NC(=N2)N(CCO)CCO)C=C1	HY-15339	MedChem Express	532.3	-1.1	-101.1	-99.9	-6.3		
NCGC00387133-01	5 -Fluoroindirubinoxime	O/N=C1\C(NC2=CC=CC=C12)=C3/C(=O)NC4=C3C=C(F)C=C4	4033	Tocris	532.3	-1.1	-80.2	-86.1	-6.3		
NCGC00389323-02	GSK-1059615	O=C1NC(=O)\C(S1)=C/C2=CC3=C(C=CN=C3C=C2)C4=CC=NC=C4	9450-5	Bio Vision	597.3	-1.2	-79.9	-87.0	-6.2		
NCGC00346992-01	Purfalcamine	NC1CCC(CC1)NC2=NC3=C(N=C[N]3C4=CC=CC(=C4)F)C(=N2)NC5=CC=C(C=C5)C(=O)N6CCCCC6	BTM012-065	NCGCChem	597.3	-1.2	-78.7	-96.3	-6.2		
NCGC00346676-09	CCT137690	CN1CCN(CC1)C2=CC=C(C=C2)C3=NC4=C(N5CCN(CC5)CC6=NOC(=C6)C)C(=CN=C4[NH]3)Br	GLXC-04300	Glixx	597.3	-1.2	-77.1	-79.3	-6.2		
NCGC00387999-01	AD57 (hydrochloride)	CC(C)[N]1N=C(C2=CC=C(NC(=O)NC3=CC(=CC=C3)C(F)(F)F)C=C2)C4=C(N)N=CN=C14	13975	Cayman	597.3	-1.1	-86.8	-79.5	-6.2		
NCGC00386728-01	NT 157	OC1=C(O)C(=CC(=C1)CNC(=S)\C=C\C2=CC(=C(O)C(=C2)Br)O)O	2238	Axon Medchem	670.2	-1.4	-60.3	-8.3	-6.2		
NCGC00387981-01	Cdk2 Inhibitor II	N[S+]([O-])(=O)C1=CC=C(N\N=C2/C(=O)NC3=CC=C(Br)C=C23)C=C1	15154	Cayman	670.2	-1.2	-67.2	-64.7	-6.2		
NCGC00346509-02	GSK1059615	O=C1NC(=O)/C(S1)=C/C2=CC=C3N=CC=C(C4=CC=NC=C4)C3=C2	S1360	Selleck	670.2	-1.1	-89.8	-91.2	-6.2		
NCGC00242057-04	GW768505A	COC1=CC=C(C=C1)C2=C(C3=CC=C(NC(=O)NC4=C(F)C=CC(=C4)C(F)(F)F)C=C3)C5=C(O2)N=CN=C5N	GLXC-03299	Glixx	670.2	-1.1	-85.9	-78.2	-6.2		
NCGC00378942-02	LY2090314	FC1=CC2=C3[N](CCN(CC3=C1)C(=O)N4CCCCC4)C=C2C/5=C(C(=O)NC5=O)\C6=CN=C7C=CC=C[N]67	S7063	Selleck	670.2	-1.1	-79.5	-83.9	-6.2		
NCGC00186035-01	IRAK-1-4 Inhibitor I	[O-][N+](=O)C1=CC=CC(=C1)C(=O)NC2=NC3=CC=CC=C3[N]2CCN4CCOCC4	Lopac-I-5409	SigmaAldrich	751.9	-1.2	-75.6	-75.2	-6.1		
NCGC00378943-01	DB07268	NC(=O)C1=CC=CC=C1NC2=NC(=NC=C2)NC3=CC=CC(=C3)O	HY-15737	MedChem Express	751.9	-1.1	-100.7	-97.2	-6.1		
NCGC00094374-13	Roscovitine	CC[C@H](CO)NC1=NC(=C2N=C[N](C(C)C)C2=N1)NCC3=CC=CC=C3	HY-30237	MedChem Express	751.9	-1.1	-100.3	-97.7	-6.1		X
NCGC00025219-10	Purvalanol A	CC(C)[C@H](CO)NC1=NC(=C2N=C[N](C(C)C)C2=N1)NC3=CC(=CC=C3)Cl	HY-18299A	MedChem Express	751.9	-1.1	-79.6	-72.7	-6.1		
NCGC00485907-01	CX-4945 (sodium salt)	[O-]C(=O)C1=CC=C2C(=C1)N=C(NC3=CC=CC(=C3)Cl)C4=C2C=NC=C4	HY-50855B	MedChem Express	843.7	-1.1	-98.0	-93.5	-6.1		
NCGC00387877-01	Voruciclib	CN1CC[C@H]([C@@H]1CO)C2=C3OC(=C/C(=O)C3=C(O)C=C2O)\C4=C(Cl)C=C(C=C4)C(F)(F)F	CT -VORU	ChemieTek	843.7	-1.1	-95.6	-95.8	-6.1		X
NCGC00384274-01	Indirubin_E8	OCC(O)CCO\N=C1\C(NC2=C1C=CC=C2)=C3\C(=O)NC4=C3C=CC=C4	402081	EMD Chemicals	843.7	-1.1	-83.1	-81.2	-6.1		
NCGC00024760-07	GF109203X	CN(C)CCC[N]1C=C(C2=CC=CC=C12)\C3=C(C(=O)NC3=O)/C4=C[NH]C5=C4C=CC=C5	S7208	Selleck	946.6	-1.2	-69.1	-74.0	-6.0		
NCGC00346645-05	TAK-901	CC[S+]([O-])(=O)C1=CC=CC(=C1)C2=CC(=C(C)C3=C2C4=C([NH]3)N=CC(=C4)C)C(=O)NC5CCN(C)CC5	HY-12201	MedChem Express	946.6	-1.1	-101.2	-96.3	-6.0		
NCGC00094244-15	SU9516	COC1=CC=C2NC(=O)C(=C\C3=CN=C[NH]3)/C2=C1	HY-18629	MedChem Express	946.6	-1.1	-93.0	-90.2	-6.0		
NCGC00386596-01	Aurora A inhibitor II	CC(=O)N1CCN(CC1)C(=O)CC2=CC=C(NC3=NC=C(F)C(=N3)NC4=CC=C(C=C4)C(=O)NC5=C(Cl)C=CC=C5)C=C2	1630	Axon Medchem	946.6	-1.1	-82.6	-83.7	-6.0		

[a] concentration at which half maximal activity is observed

[b] criteria to classify curves based on the quality of curve fit to the data, the magnitude of the response (efficacy), and the number of asymptotes to the calculated curve

[c] magnitude of response calculated as difference between highest and lowest activity measures across concentration range
